# An explainable artificial intelligence-enabled electrocardiogram analysis model for the classification of reduced left ventricular function

**DOI:** 10.1093/ehjdh/ztad027

**Published:** 2023-04-17

**Authors:** Susumu Katsushika, Satoshi Kodera, Shinnosuke Sawano, Hiroki Shinohara, Naoto Setoguchi, Kengo Tanabe, Yasutomi Higashikuni, Norifumi Takeda, Katsuhito Fujiu, Masao Daimon, Hiroshi Akazawa, Hiroyuki Morita, Issei Komuro

**Affiliations:** Department of Cardiovascular Medicine, The University of Tokyo Hospital, 7-3-1 Hongo, Bunkyo-ku, Tokyo 113-8655, Japan; Department of Cardiovascular Medicine, The University of Tokyo Hospital, 7-3-1 Hongo, Bunkyo-ku, Tokyo 113-8655, Japan; Department of Cardiovascular Medicine, The University of Tokyo Hospital, 7-3-1 Hongo, Bunkyo-ku, Tokyo 113-8655, Japan; Department of Cardiovascular Medicine, The University of Tokyo Hospital, 7-3-1 Hongo, Bunkyo-ku, Tokyo 113-8655, Japan; Department of Cardiovascular Medicine, Mitsui Memorial Hospital, 1 Kanda-Izumi-cho, Chiyoda-ku, Tokyo 101-8643, Japan; Department of Cardiovascular Medicine, Mitsui Memorial Hospital, 1 Kanda-Izumi-cho, Chiyoda-ku, Tokyo 101-8643, Japan; Department of Cardiovascular Medicine, The University of Tokyo Hospital, 7-3-1 Hongo, Bunkyo-ku, Tokyo 113-8655, Japan; Department of Cardiovascular Medicine, The University of Tokyo Hospital, 7-3-1 Hongo, Bunkyo-ku, Tokyo 113-8655, Japan; Department of Advanced Cardiology, The University of Tokyo, 7-3-1 Hongo, Bunkyo-ku, Tokyo 113-8655, Japan; Department of Clinical Laboratory, The University of Tokyo Hospital, 7-3-1 Hongo, Bunkyo-ku, Tokyo 113-8655, Japan; Department of Cardiovascular Medicine, The University of Tokyo Hospital, 7-3-1 Hongo, Bunkyo-ku, Tokyo 113-8655, Japan; Department of Cardiovascular Medicine, The University of Tokyo Hospital, 7-3-1 Hongo, Bunkyo-ku, Tokyo 113-8655, Japan; Department of Cardiovascular Medicine, The University of Tokyo Hospital, 7-3-1 Hongo, Bunkyo-ku, Tokyo 113-8655, Japan

**Keywords:** Explainable Artificial intelligence, Artificial intelligence, Machine learning, Electrocardiogram, Echocardiography, Left ventricular dysfunction

## Abstract

**Aims:**

The black box nature of artificial intelligence (AI) hinders the development of interpretable AI models that are applicable in clinical practice. We aimed to develop an AI model for classifying patients of reduced left ventricular ejection fraction (LVEF) from 12-lead electrocardiograms (ECG) with the decision-interpretability.

**Methods and results:**

We acquired paired ECG and echocardiography datasets from the central and co-operative institutions. For the central institution dataset, a random forest model was trained to identify patients with reduced LVEF among 29 907 ECGs. Shapley additive explanations were applied to 7196 ECGs. To extract the model’s decision criteria, the calculated Shapley additive explanations values were clustered for 192 non-paced rhythm patients in which reduced LVEF was predicted. Although the extracted criteria were different for each cluster, these criteria generally comprised a combination of six ECG findings: negative T-wave inversion in I/V5–6 leads, low voltage in I/II/V4–6 leads, Q wave in V3–6 leads, ventricular activation time prolongation in I/V5–6 leads, S-wave prolongation in V2–3 leads, and corrected QT interval prolongation. Similarly, for the co-operative institution dataset, the extracted criteria comprised a combination of the same six ECG findings. Furthermore, the accuracy of seven cardiologists’ ECG readings improved significantly after watching a video explaining the interpretation of these criteria (before, 62.9% ± 3.9% vs. after, 73.9% ± 2.4%; *P* = 0.02).

**Conclusion:**

We visually interpreted the model’s decision criteria to evaluate its validity, thereby developing a model that provided the decision-interpretability required for clinical application.

## Graphical Abstract

**Graphical Abstract:**
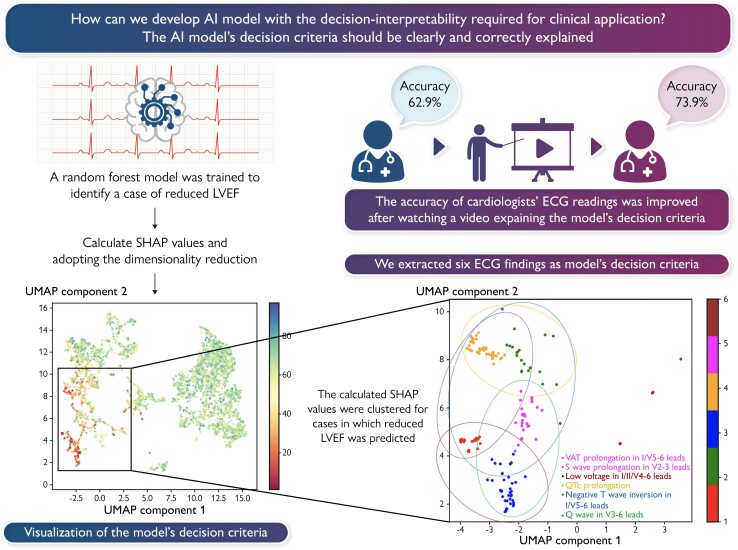

## Introduction

In recent years, artificial intelligence (AI) has made remarkable progress in medicine. Various clinical studies on AI have been reported and implemented in clinical practice.^1–7^ In daily practice, physicians provide medical care by interpreting a great deal of information, including medical history, physical examination findings, blood tests, imaging tests, the patient’s social background, and treatment guidelines. AI can also help physicians’ clinical decisions. From this perspective, AI models implemented in medical practice should achieve both high-performance and considerable interpretability.^7,8^ However, many AI models involve a trade-off between high accuracy and high interpretability.^9^ This so-called ‘black box’ problem with AI is seen not only in the medical field, but also in many other fields. Therefore, various machine learning methods have been developed to increase the ‘explainability’ of AI models, which are known as explainable AI (XAI).^10^ XAIs can show which parts of the input data have a strong influence on the predicted outcomes. Although the physicians’ needs for interpretable AI models might be to understand the causality between the input data and predicted outcomes within the AI model, XAI does not reveal this causality; that is, what findings in the input data are deduced to lead to the predicted results. Unless the causality is clarified, it is likely to be difficult for physicians to apply the predicted outcomes provided by the XAI in clinical practice. Therefore, the current applications of XAI alone cannot satisfy physicians’ needs for interpretable AI models.^11^

We previously developed a convolutional neural network-based AI model to classify patients with reduced left ventricular ejection fraction (LVEF) from raw 12-lead electrocardiogram (ECG) data. The performance of this model was shown by an area under the receiver operating characteristic curve (AUROC) of 0.945.^12^ However, it was unclear what ECG findings the model used to derive the predicted results. Accordingly, if this model was to be implemented in the medical field, physicians would make insufficient clinical decisions when interpreting the model’s predicted results because they would not have a benchmark to use as a reference. Therefore, we aimed to develop an AI model for classifying patients of reduced LVEF from 12-lead ECG data with sufficient interpretability to satisfy physicians’ needs when the model is implemented in clinical practice.

## Methods

### Study sample

**Figure 1:**
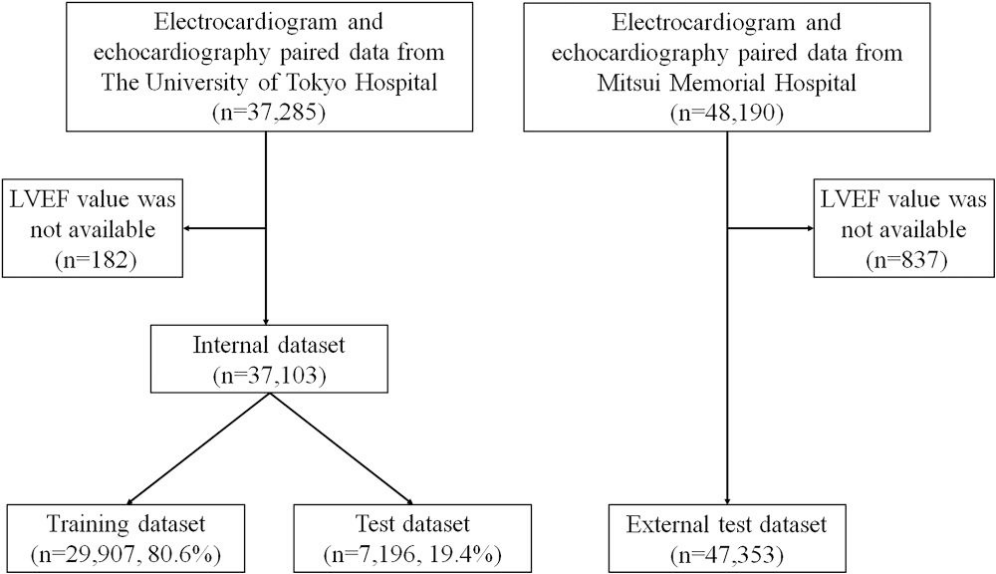
Data flow. Flowchart showing how the three datasets used for model training, evaluation and external validation were created. To avoid cross-contamination across the training and test datasets included the internal dataset, multiple data pairs from a single patient were included only within the same dataset. LVEF, left ventricular ejection fraction.

As in the previous study,^12^ this study used data from patients aged 18 years or older who underwent echocardiography at The University of Tokyo Hospital between January 2015 and December 2019 and had an ECG performed within 28 days of their echocardiography. The ECG and matched echocardiography data were paired (one-to-one correspondence between the ECG and the echocardiography), and 37 103 sets of paired data (the internal dataset) were randomly divided into a training dataset (29 907 pairs from 15 135 patients; 80.6%) and test dataset (7196 pairs from 3784 patients; 19.4%). Patients with multiple paired data were included in the same dataset (*Figure 1*). Similarly, we collected data from patients aged 18 years or older who underwent echocardiography at Mitsui Memorial Hospital, and 47 353 sets of paired data were used as an external test dataset (*Figure 1*). The study was conducted in accordance with the revised Declaration of Helsinki and was approved by the Institutional Review Board of The University of Tokyo [reference number: 2021132NI-(2)]. Informed consent was obtained in the form of an opt-out on a website.

### Electrocardiography and echocardiography data acquisition

ECGs were recorded for a 10-s interval at a sampling rate of 500 Hz using an FCP-8700 or FCP-8800 system (Fukuda Denshi, Tokyo, Japan). ECG parameters were obtained through automatic analysis of the ECGs. The ECG parameters are shown in Supplementary material online, *Table S1*. Echocardiography was performed by skilled sonographers or cardiologists. Echocardiographic measurements were obtained in accordance with the American Society of Echocardiography recommendations at the time of acquisition,^13^ and each echocardiography was interpreted by one or two experienced echocardiologists. Reduced LVEF was defined as an ejection fraction of <40%.^14^

### Model development

Of the ECG parameters acquired, 178 parameters were used as input data (see Supplementary material online, *Table S1*). Then, we trained several machine learning and deep learning models—support vector machine model,^15^ logistic regression model,^16^ random forest model,^17^ and multi-layer perceptron model^18^—to classify whether a patient had reduced LVEF, using only the data included in the training dataset. Among these models, we extracted the model’s decision criteria for the model that performed best in the validation of the test dataset. These models were constructed in the Python language using the Scikit-learn machine learning library (https://scikit-learn.org/stable/about.html#citing-scikit-learn) and the PyTorch deep learning library (https://pytorch.org).

### Adaptation and interpretation of XAI

The model’s decision criteria needed to be interpreted in a two-stage fashion. First, the contribution of each ECG parameter to the model’s classification of patients with reduced LVEF needed to be calculated. Shapley additive explanations (SHAP)^19,20^ were used to compute this contribution for the test dataset. SHAP involves decomposing the difference between the expected predictions of the model and the obtained predictions as the contribution of each element of the input data. In calculating this contribution, the Shapley value of co-operative game theory is applied to calculate the average marginal contribution of each input element as an SHAP value.^19^ Second, the model’s decision criteria for patients with reduced LVEF were then clarified by performing pattern classification based on the calculated SHAP value. However, the calculated SHAP value represented 178-dimensional information per ECG, and needed dimension reduction to perform pattern classification with high accuracy. Hence, the calculated SHAP values were reduced to two dimensions using the principal component analysis (PCA)–uniform manifold approximation and projection (UMAP) method,^21^ which combines PCA^22^ and UMAP,^23^ a dimensionality reduction method. Then, the 192 ECGs included in the test dataset in which reduced LVEF was predicted, excluding those with a paced rhythm, were clustered by adapting a variational Bayesian Gaussian mixture model (VBGMM)^24^ to the two-dimensional SHAP values. When the model’s predicted value exceeded a cut-off of 0.5, reduced LVEF was predicted in that patient.

### Explanation of the developed model’s decision criteria

On the basis of the relationship between the SHAP values and ECG parameters for each classified cluster, the model’s decision criteria for patients with reduced LVEF were explained. First, if the median SHAP value for each ECG parameter was greater than the mean + standard deviation of the SHAP values for all ECG parameters, that ECG parameter was defined as a decision factor; i.e. a factor influencing the model’s decision to classify the ECG as a patient with reduced LVEF. Next, on the basis of the distribution of the actual ECG parameters and SHAP values identified as decision factors, ECG findings that provided the basis of the model’s determinations of patients with reduced LVEF were extracted as the model’s decision criteria.

### Evaluation of the models’ performance and validity of the model’s decision criteria

The diagnostic performance of the models was validated on the test dataset by calculating accuracy, sensitivity, specificity and AUROC with the output cut-off value set to 0.5. Conventional ECG interpretation was also performed to assess the validity of the model’s decision criteria. Seven board-certified cardiologists, each with over 8 years of clinical experience, participated in this ECG interpretation test. First, 100 ECGs (50% of patients with reduced LVEF) were randomly selected from the test dataset while avoiding selecting the same patient several times and excluding ECGs with a paced rhythm. Second, each cardiologist independently read the 100 ECGs for the presence or the absence of reduced LVEF. Decisions during ECG reading were made on the basis of the impression of each cardiologist because there are no established ECG criteria for predicting the presence of reduced LVEF. Finally, after watching a video explaining the results of this study (educational video), each cardiologist read the same 100 randomly sorted ECGs for the presence or the absence of reduced LVEF. The accuracy, sensitivity and specificity of the seven cardiologists’ interpretations before watching the educational video were compared with those after watching it.

### External validation of the model’s decision criteria

The same explanations were also performed on the external test dataset to validate the generalizability of the extracted model’s decision criteria. The 1244 ECGs included in the external test dataset in which reduced LVEF was predicted, excluding those with a paced rhythm, were used to explain the model’s decision criteria.

### Statistical analysis

Continuous variables are presented as mean and standard deviation and were compared using unpaired Student’s *t*-tests. Categorical variables are expressed as numbers and percentages and were compared using χ^2^ tests. The 95% confidence intervals (CIs) of accuracy, sensitivity, specificity, and AUROC were calculated using bootstrapping (resampling 10 000 times with replacement).^25^ Obuchowski’s method was used to evaluate the educational effectiveness of the ECG interpretation test.^26^ This extends the McNemar test to a situation where the observations are sampled in clusters. Statistical analysis was performed using R version 4.1.1 (clust.bin.pair-package authorized by Dan Gopstein; www.r-project.org), and statistical significance was defined as a *P*-value of <0.05.

## Results

### Patient characteristics

The internal dataset comprised 37 103 ECG–echocardiography pairs from 18 919 patients. The median period between the acquisition of the paired ECG and echocardiography was 1 day. The external test dataset comprised 47 353 ECG–echocardiography pairs from 23 473 patients. The median period between the acquisition of the paired ECG and echocardiography was 1 day. The characteristics of the patients in the internal dataset and the external test dataset are shown in *Table 1* and Supplementary material online, *Table S2*, respectively. The mean age in the internal dataset and the external test dataset was 63.4 ± 16.9 and 69.8 ± 13.9 years, respectively. There were 21 025 ECGs from 10 403 men and 16 078 ECGs from 8516 women in the internal dataset and 29 805 ECGs from 14 141 men and 17 548 ECGs from 9332 women in the external test dataset. We enrolled 3501 ECGs from 1116 patients with reduced LVEF in the internal dataset and 4187 ECGs from 1667 patients with reduced LVEF in the external test dataset. The training and test datasets comprised 29 907 ECGs from 15 135 patients (80.6%) and 7196 ECGs from 3784 patients (19.4%), respectively (*Figure 1* and *Table 1*). The distributions of patients with reduced LVEF in each dataset are shown in *Table 1*.

**Table 1:** Patient characteristics in the internal dataset at the time of ECG and echocardiogram acquisition

	Training dataset (*n* = 29 907)	Test dataset (*n* = 7196)	*P*-value
Age (years)	63.3 (17.0)	63.5 (16.7)	0.295
Male, *n* (%)	16 886 (56.5)	4139 (57.5)	0.107
Body height (cm)	161.7 (9.9)	161.8 (10.0)	0.808
Body weight (kg)	59.9 (13.6)	60.3 (13.6)	0.068
LVEF (%)	61.2 (14.7)	60.9 (15.2)	0.064
Reduced LVEF, *n* (%)	2764 (9.2)	737 (10.2)	0.010
HR (/min)	74.0 (15.4)	74.1 (15.1)	0.543
PR interval (ms)	171.5 (43.9)	169.9 (43.9)	0.007
QRS duration (ms)	105.8 (22.6)	106.1 (22.6)	0.232
QT interval (ms)	396.9 (40.0)	396.9 (39.3)	0.956
QTc	435.0 (34.1)	435.6 (35.0)	0.142
QRS axis	28.1 (45.3)	28.5 (44.5)	0.429
P axis	41.5 (34.8)	41.6 (33.9)	0.782

Data are presented as *n* (%) or mean (standard deviation). *P*-values are from unpaired Student’s t-tests or the χ^2^ test and indicate differences in the distribution of values between the training and test datasets.

ECG, electrocardiogram; LVEF, left ventricular ejection fraction; HR, heart rate; QTc, corrected QT interval.

### Diagnostic performances

**Figure 2:**
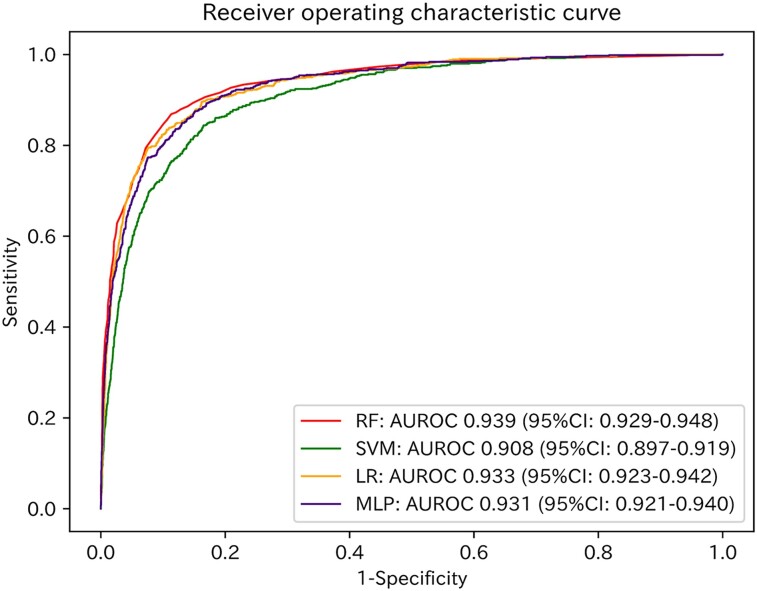
Receiver operating characteristic curves of the four models applied to the test dataset. Red, green, orange, and purple lines represent the receiver operating characteristic curves of the random forest model, support vector machine model, logistic regression model, and multi-layer perceptron model, respectively. RF, random forest model; SVM, support vector machine model; LR, logistic regression model; MLP, multi-layer perceptron model; AUROC, area under the receiver operating characteristic curve; CI, confidence interval.

The AUROC values of the models for the test dataset are shown in *Figure 2*. The model with the best performance was the random forest model (AUROC: 0.939; 95% CI: 0.929–0.948; *Figure 2*). With a cut-off value of 0.5, the accuracies, sensitivities, and specificities of the models for the test dataset are shown in Supplementary material online, *Table S3*.

### Adapting SHAP in each model

By adapting SHAP to the four trained models, we extracted the ECG parameters that contributed significantly to each model’s decision across the test dataset (see Supplementary material online, *Figures S1–S4*). Although there were differences in the extracted ECG parameters in each model, most were components of QRS duration, R amplitude, and QT interval.

### Visualization and clustering of the SHAP values for the test dataset

**Figure 3:**
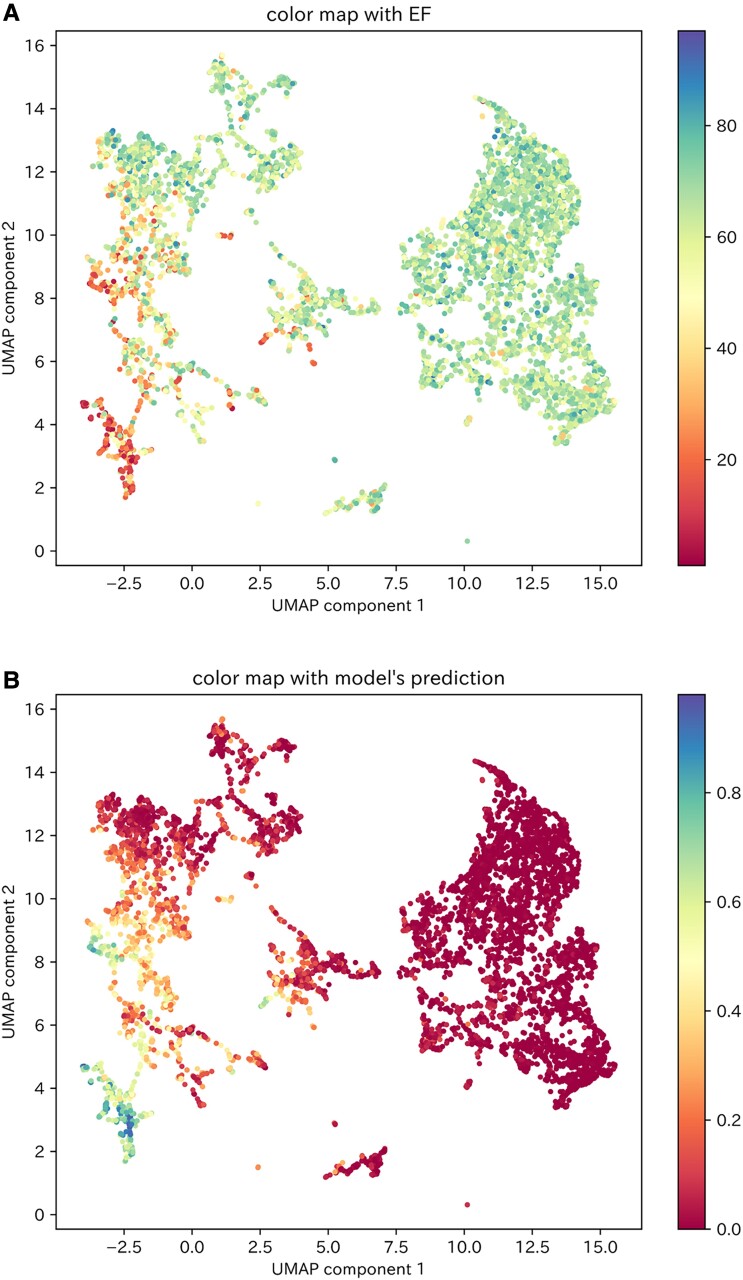
Visualization of the two-dimensional SHAP values of the test dataset. (*A*) Colour mapping of the two-dimensional SHAP values of the test dataset according to the LVEF values of each data item. (*B*) Colour mapping of the two-dimensional SHAP values of the test dataset according to the model’s predictive values for each data item. UMAP, uniform manifold approximation and projection.

**Figure 4:**
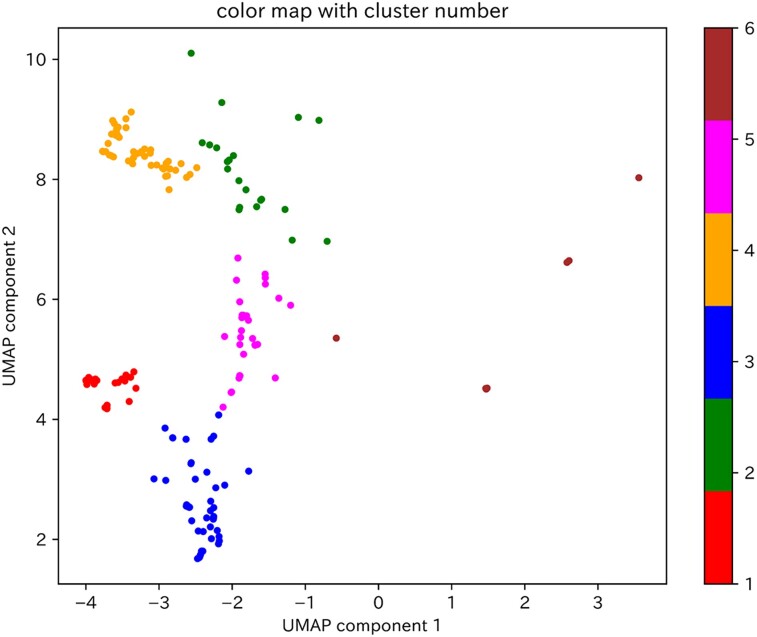
Clustering of two-dimensional SHAP values for ECGs in the test dataset by which the model predicted reduced LVEF. The 192 ECGs by which the model predicted reduced LVEF, excluding those with a paced rhythm, were clustered by adapting a variational Bayesian Gaussian mixture model to the two-dimensional SHAP values. The clusters are classified from 1 to 6, and the data points belonging to each cluster are coloured red, green, blue, orange, pink, or brown. UMAP, uniform manifold approximation and projection.

Two-dimensional visualization of the calculated SHAP values for the test dataset using the PCA–UMAP method is shown in *Figure 3*. This visualization suggests that the ECGs for which the model gave a high predictive value could be divided into multiple clusters. In other words, the model had multiple criteria for determining patients of reduced LVEF. Then, of the 429 ECGs in the test dataset in which the model predicted reduced LVEF, 192 (excluding 237 ECGs with a paced rhythm) were classified into one of six clusters using the VBGMM for the two-dimensional SHAP values (*Figure 4*). The characteristics of the ECG data included in each cluster are shown in Supplementary material online, *Table S4*.

### Interpretation of the model’s decision criteria

**Figure 5:**
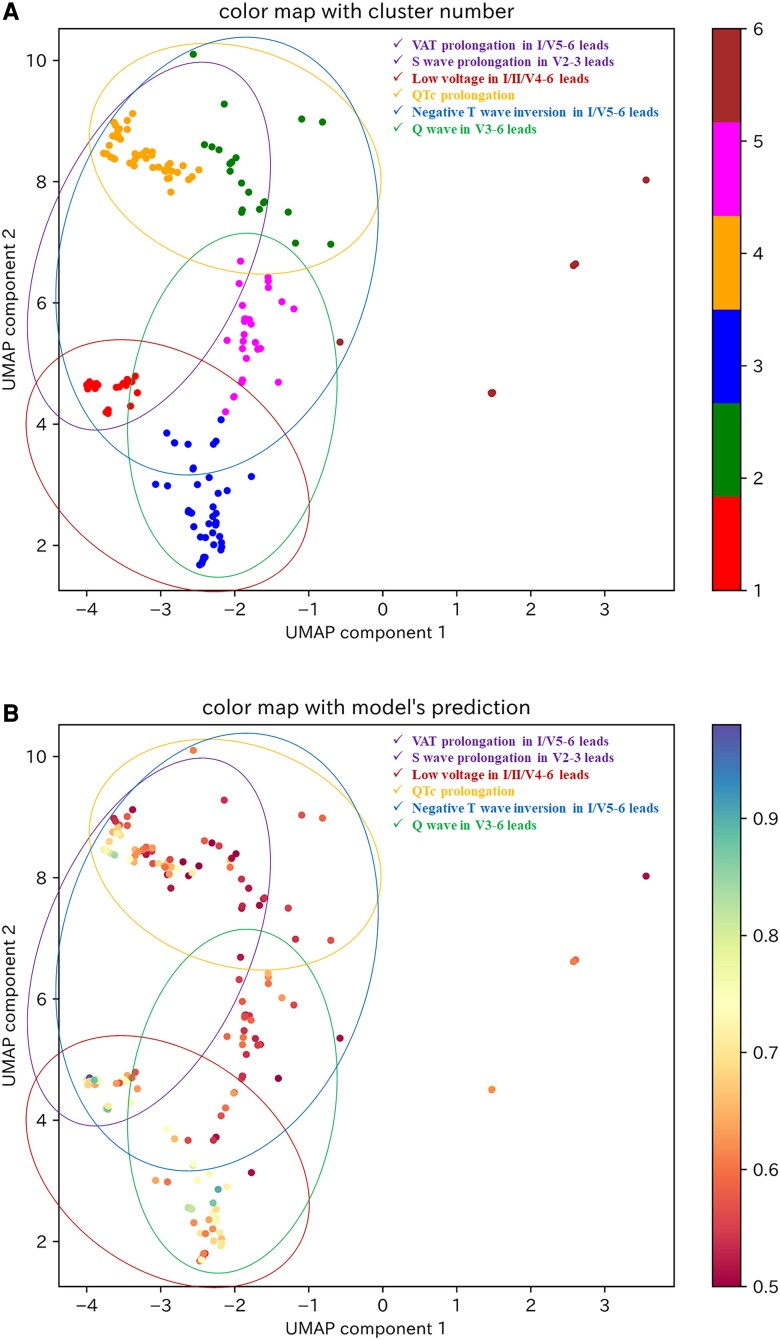
The six categories of ECG findings on a visualization of the two-dimensional SHAP values for ECGs to predict reduced LVEF in the test dataset. (*A*) The six categories of ECG findings on a visualization of the clustered two-dimensional SHAP values for ECGs to predict reduced LVEF. (*B*) The six categories of ECG findings on colour mapped two-dimensional SHAP values by the model’s predictive value in ECGs to predict reduced LVEF. The purple circle represents the category of VAT prolongation in I/V5–6 leads and S-wave prolongation in V2–3 leads. The brown circle represents the category of low voltage in I/II/V4–6 leads. The orange circle represents the category of QTc prolongation. The blue circle represents the category of negative T-wave inversion in I/V5–6 leads. The green circle represents the category of Q wave in V3–6 leads. UMAP, uniform manifold approximation and projection; VAT, ventricular activation time; QTc, corrected QT interval.

As there were nine ECGs included in Cluster 6, which we considered to be an insufficient number for explanation of the model’s decision criteria, we investigated the model’s decision criteria for Clusters 1–5. The decision factors for each cluster are shown in *Table 2* and Supplementary material online, *Figures S5–S9*, and the model’s decision criteria, which were interpreted from the relationship between the extracted decision factors and SHAP values (see Supplementary material online, *Figures S10–S14*), are shown in *Table 2*. Although the decision criteria in each cluster were different, they were generally composed of a combination of six ECG findings: negative T-wave inversion in I/V5–6 leads, low voltage in I/II/V4–6 leads, Q wave in V3–6 leads, ventricular activation time (VAT) prolongation in I/V5–6 leads, S-wave prolongation in V2–3 leads, and corrected QT interval (QTc) prolongation (*Table 3*). Findings of low voltage in I/II/V4–6 leads, negative T-wave inversion in I/V5–6 leads, and S-wave prolongation in V2–3 leads overlapped between several clusters, particularly Clusters 1, 3, and 4, which showed high model predictive values (see Supplementary material online, *Table S4*). The categories of these ECG findings are displayed on the visualization of the two-dimensional SHAP values shown in *Figure 5*. As VAT prolongation in I/V5–6 leads and S-wave prolongation in V2–3 leads were related to intraventricular conduction delay, these parameters were considered to be within the same category. We suggest that a category related to intraventricular conduction delay and a category showing low voltage in a broad range of leads may be strongly associated with a high model predictive value (*Figure 5B*).

**Table 2:** Decision factors and decision criteria in each cluster

	Decision factors	Decision criteria
Cluster 1	T-wave amp. in I/aVR/V5–6 leads R-wave amp. in II/V4–5 leads Age S-wave dur. in V3 lead	Negative T-wave inversion in I/V5–6 leads Low voltage in II/V4–5 leads S-wave prolongation in V3 lead
Cluster 2	T-wave amp. in I/II/aVR/V5–6 leads VAT in V5–6 leads R-wave amp. in V4 lead QTc in V1–3 leads S-wave dur. in V3 lead	Negative T-wave inversion in I/II/V5–6 leads VAT prolongation in V5–6 leads Low voltage in V4 lead QTc prolongation in V1–3 leads S-wave prolongation in V3 lead
Cluster 3	R wave amp. in I/II/V4–6 leads Q-wave dur. in V4–6 leads R-wave dur. in V5 lead Age VAT in V4 lead	Low voltage in I/II/V4–6 leads Q-wave in V4–6 leads
Cluster 4	T wave amp. in I/aVR/V5–6 leads VAT in I/V6 leads QTc in III/aVL/V1–5 leads S-wave dur. in V2–3 leads Age	Negative T-wave inversion in I/V5–6 leads VAT prolongation in I/V6 leads QTc prolongation in III/aVL/V1–5 leads S wave prolongation in V2–3 leads
Cluster 5	T wave amp. in I/II/aVR/V5–6 leads R wave amp. in II/V4 leads Q wave dur. in V3–4 leads VAT in V4 lead R wave dur. in V3–4 leads Q wave amp. in V3 lead	Negative T inversion in I/II/V5–6 leads Low voltage in II/V4 leads Q wave in V3–4 leads

Decision factors are defined as the ECG parameters that influenced the model’s decision that the ECG was a patient of reduced left ventricular ejection fraction. Decision criteria are ECG findings that contributed to the model’s decision, which are interpreted from the relationship between the decision factors and SHAP values.

ECG, electrocardiogram; amp., amplitude; dur., duration; VAT, ventricular activation time; QTc, corrected QT interval.

**Table 3:** Combinations of the six ECG categorizes in each cluster

	ECG findings
Cluster 1	Negative T-wave inversion in I/V5–6 leads Low voltage in I/II/V4–6 leads S wave prolongation in V2–3 leads
Cluster 2	Negative T-wave inversion in I/V5–6 leads VAT prolongation in I/V5–6 leads S wave prolongation in V2–3 leads QTc prolongation
Cluster 3	Low voltage in I/II/V4–6 leads Q wave in V3–6 leads
Cluster 4	Negative T-wave inversion in I/V5–6 leads VAT prolongation in I/V5–6 leads S wave prolongation in V2–3 leads QTc prolongation
Cluster 5	Negative T-wave inversion in I/V5–6 leads Low voltage in I/II/V4–6 leads Q wave in V3–6 leads

The decision criteria extracted for each cluster could be decomposed into six ECG findings. Data are presented for the combinations of the six ECG findings in each cluster.

VAT, ventricular activation time; QTc, corrected QT interval.

### Assessment of the validity of the interpretable model’s decision criteria

The cardiologists’ diagnostic performance values in the ECG interpretation before and after viewing the educational video are shown in *Table 4*. Before viewing the video, the mean accuracy, sensitivity, and specificity were 62.9% ± 3.9%, 37.4% ± 9.3%, and 88.3 ± 7.1%, respectively. After viewing the video, the mean accuracy, sensitivity, and specificity were 73.9% ± 2.4%, 71.1% ± 8.1%, and 76.6% ± 8.8%, respectively, with significant improvements in accuracy and sensitivity (*Table 4*; both *P* = 0.02).

**Table 4:** Cardiologists’ diagnostic performance in the ECG interpretation test

	Before watching educational video			After watching educational video		
	Accuracy	Sensitivity	Specificity	Accuracy	Sensitivity	Specificity
Cardiologist 1	62.0	52.0	72.0	72.0	66.0	78.0
Cardiologist 2	57.0	26.0	88.0	76.0	70.0	82.0
Cardiologist 3	64.0	36.0	92.0	71.0	84.0	58.0
Cardiologist 4	63.0	36.0	90.0	74.0	60.0	88.0
Cardiologist 5	61.0	34.0	88.0	71.0	64.0	78.0
Cardiologist 6	71.0	50.0	92.0	77.0	74.0	80.0
Cardiologist 7	62.0	28.0	96.0	76.0	80.0	72.0
Mean (SD)	62.9 (3.9)*	37.4 (9.3)**	88.3 (7.1)	73.9 (2.4)*	71.1 (8.1)**	76.6 (8.8)

The cardiologists’ diagnostic performances before or after watching the educational video are shown as percentages. The means and standard deviations (SD) are also shown.

*Interaction between the accuracy of the cardiologists’ interpretations before and after watching the educational video; *P* = 0.02.

**Interaction between the sensitivity of the cardiologists’ interpretations before and after watching the educational video; *P* = 0.02.

### External validation of the model’s decision criteria

The AUROC value of the random forest model for the external test dataset is shown in Supplementary material online, *Figure S15* (AUROC: 0.908; 95% CI: 0.904–0.912). Two-dimensional visualization of the calculated SHAP values for the external test dataset using the PCA–UMAP method is shown in Supplementary material online, *Figures S16* and *S17*. This visualization suggests that the distribution of the model’s decision criteria was similar in the test dataset and the external test dataset. Then, of the 1876 ECGs in the test dataset in which the model predicted reduced LVEF, 1244 ECGs (excluding 632 ECGs with a paced rhythm) were classified into one of seven clusters using the VBGMM for the two-dimensional SHAP values (see Supplementary material online, *Figure S18*). The characteristics of the ECG data in each cluster are shown in Supplementary material online, *Table S5*. The decision factors for each cluster are shown in Supplementary material online, *Table S6* and *Figures S19–S25*. The model’s decision criteria, which were interpreted from the relationship between the extracted decision factors and the SHAP values (see Supplementary material online, *Figures S26–S32*), are shown in Supplementary material online, *Table S6*. As in the test dataset, the model’s decision criteria extracted in the external test dataset could be expressed as a combination of the six ECG categories (see Supplementary material online, *Table S7* and *Figure S33*).

## Discussion

In this study, we used SHAP, dimensionality reduction, and cluster analysis to explain the decision criteria used by an AI model that accurately classifies patients with reduced LVEF from their ECG data. We also created an educational video on the model’s decision criteria and evaluated their validity with an ECG interpretation test. Furthermore, we also validated the generalizability of the model’s decision criteria using external validation data. As a result, we were able to develop an AI model that might satisfy the needs of the decision-interpretability required in clinical applications.

In a previous study comparing ECG findings between normal subjects and patients with heart failure,^27^ QRS prolongation, VAT prolongation in V5–6 leads, axis deviation, QT prolongation, left ventricular hypertrophy, ST-T abnormalities, and left bundle branch block were identified as ECG findings associated with heart failure with reduced LVEF (HFrEF), whereas increased resting heart rate, P-wave axis abnormalities and QRS-T axis abnormalities were identified as ECG findings associated with heart failure with preserved LVEF (HFpEF). In addition, a comparison of ECG findings between HFrEF and HFpEF showed that VAT prolongation in V5–6 leads, QT prolongation, and ST-T abnormalities were extracted as ECG findings associated with HFrEF. In the present study, the model’s decision criteria included VAT prolongation in I/V5–6 leads, negative T-wave inversion in I/V5–6 leads, and QTc prolongation, which overlap with the findings of the previous study.^27^ In this regard, the interpretable model’s decision criteria seem reasonable as ECG findings in patients with reduced LVEF.

**Figure 6:**
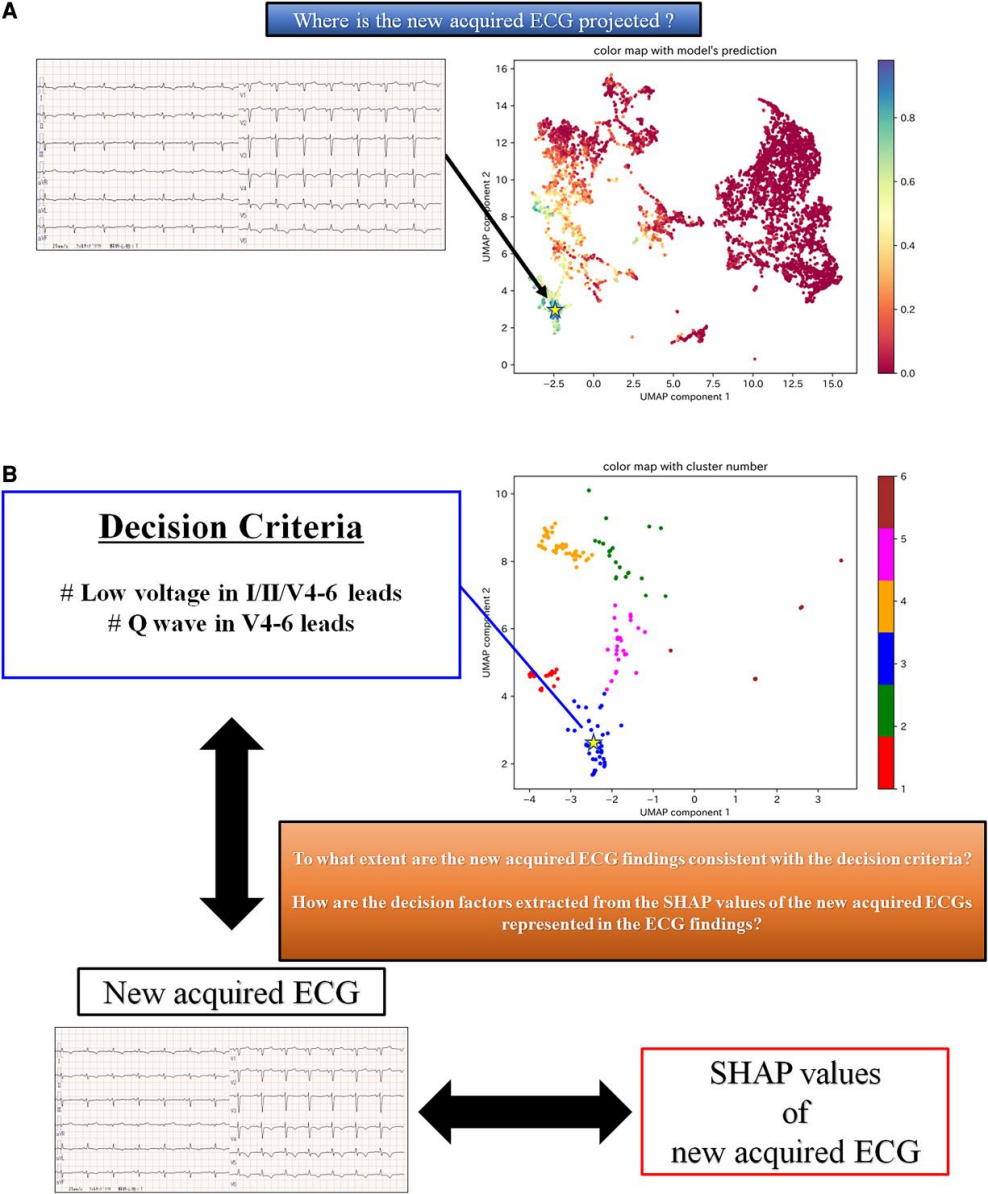
An example of how our model could be used in clinical practice, focusing on interpretability. (*A*) This figure shows where the two-dimensional SHAP values of a newly acquired ECG are projected onto the colour-mapping of the two-dimensional SHAP values for the test dataset according to the LVEF values of each data item. For example, in this figure, the newly acquired ECG is projected onto the location indicated by a star. We can approximately identify the LVEF values of patients with similar decision criteria to the newly acquired ECG. (*B*) This figure shows how to use the clustered two-dimensional SHAP values and the SHAP values of the newly acquired ECG when the model predicts that the newly acquired ECG is a reduced LVEF patient. For example, if the newly acquired ECG is projected to a star position in the clustered two-dimensional SHAP values, it can be recognized to which cluster it is likely to belong. From the SHAP values of the newly acquired ECG, decision factors indicating the ECG parameters that the model considers important for this ECG can be extracted. By referring to the interpreted decision criteria in the cluster and the extracted decision factors of the newly acquired ECG, we may be able to understand the ECG findings that seem to indicate reduced LVEF, as expressed in the newly acquired ECG. UMAP, uniform manifold approximation and projection; SHAP, Shapley additive explanations.

An example of how the interpretability of the AI model could be presented to physicians in clinical practice is shown in *Figure 6*. By reflecting the SHAP values of the ECGs in the existing two-dimensional space, we can approximately identify the LVEF values of patients with similar decision criteria (*Figure 6A*). As a result, we could determine the confidence level for the predictive results of the AI model. When the AI model predicts reduced LVEF, the clustered two-dimensional space can help us recognize which cluster the ECG belongs to. By referring to the decision criteria indicated in the cluster and the SHAP value of the ECG, the physician can compare the AI model’s decision criteria with the actual ECG findings, which should assist in decision making (*Figure 6B*).

Adapting XAI to AI models not only contributes to improved interpretability after implementation, but it may also lead to the discovery of new findings.^11,28^ The finding of S-wave prolongation in V2–3 leads, as identified in this study, was not included in the analysis in a previous study on ECG findings in patients with HFrEF.^27,29^ In the present study, we performed a comprehensive analysis using many ECG parameters previously considered unimportant. As a result, we were able to show that S-wave prolongation in V2–3 leads may be an important new ECG criterion in patients of reduced LVEF.

Furthermore, we showed that educating cardiologists on the decision criteria of the AI model contributed to improvement in their ECG reading accuracy. Although there are reports of improved diagnostic ability by ‘referencing’ AI,^12,30^ there are no reports on improved diagnostic ability by ‘learning’ from AI. Even if AI becomes more widely used in medical practice in the future, it is not always possible to use the AI models that satisfy physicians’ needs. In such a case, a physician’s medical skills are obviously important. Hence, physicians must always strive to improve their medical practice capabilities. This study has presented the possibility of a new AI–physician relationship, in which the physician does not just ‘use’ a qualified AI model, but also ‘learns’ from it.

This study has several limitations. First, the ECG–echocardiography data pairs were not acquired simultaneously, with a slight temporal delay between the components of the paired data. However, this temporal delay was small, with both assessments being obtained within a few days for most pairs. Second, the number of patients available for interpretation of the model’s decision criteria was limited. As the ECG patterns of patients with reduced LVEF may not have been analysed comprehensively, further analysis using more data is needed. Finally, the explanation evaluated in this study has not been applied to a deep learning model. Many deep learning models are now being applied in clinical research and implemented in medical practice. Technological improvements in deep learning models and their interpretability are desired.

## Conclusion

In this study, we succeeded in using XAI to objectively interpret the decision criteria of an AI model applied to ECG analysis and evaluated its validity. We were able to develop a model that could satisfy the interpretability required for clinical application.

## Lead author biography


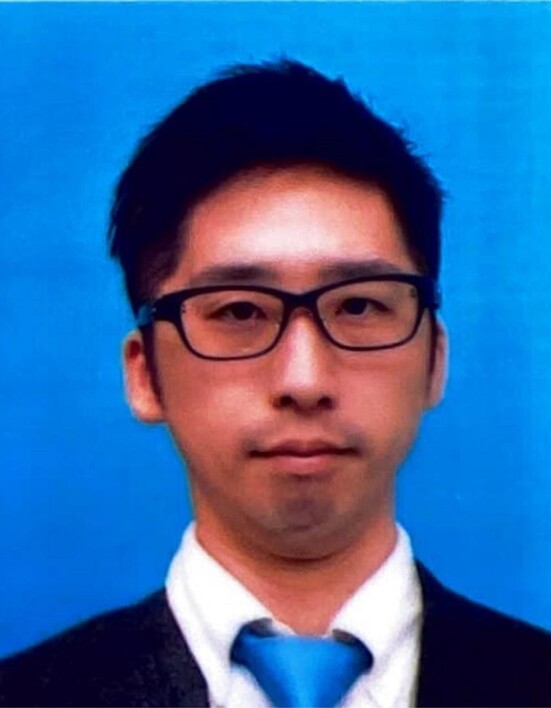
Dr Susumu Katsushika is a 10th year cardiologist. After training in general cardiovascular medicine, especially interventions for ischaemic heart disease, as a cardiovascular fellow at NTT Medical Center Tokyo, he has been a member of the Department of Cardiovascular Medicine, The University of Tokyo Hospital since 2019. His research interest is the application of artificial intelligence to cardiovascular practice.

## Supplementary material

Supplementary material is available at *European Heart Journal – Digital Health* online.

## Supplementary Material

ztad027_Supplementary_Data

## Data Availability

In the informed consent obtained in the form of an opt-out on a website, participants were informed that data from this study would not be shared with other researchers, even if the individuals were not identifiable. Therefore, we regret that the data used in this study cannot be shared. Sample code for all data processing and analysis presented in this work is available on request (contact the corresponding author).
