# Supplementary material for: An explainable artificial intelligence-enabled electrocardiogram analysis model for the classification of reduced left ventricular function
Source: Eur Heart J Digit Health. 2023 Apr 17;4(3):254–64. doi: 10.1093/ehjdh/ztad027 (PMC10232279; doi:10.1093/ehjdh/ztad027)
Supplement: ztad027_Supplementary_Data [file ztad027_supplementary_data.docx]

**Supplementary Table 1. Collected ECG parameters and input data.**

|  | ECG parameters |
| --- | --- |
| The collected ECG parameters | Age, Sex, Heart rate, RR interval, PR interval, QRS duration, QT interval, QTc, QRS axis, P axis  The following parameters were collected from each lead:  P wave amplitude and duration, Q wave amplitude and duration,  R wave amplitude and duration, S wave amplitude and duration,  R’ wave amplitude and duration, J wave amplitude and duration,  STJ, ST1, ST2, ST3, T wave amplitude, QT interval, QTc,  PR interval, QRS duration, VAT |
| The ECG parameters used as the input data | Age, Sex, Heart rate, RR interval, PR interval, QRS duration, QT interval, QTc, QRS axis, P axis  The following parameters were collected from each lead:  Q wave amplitude and duration, R wave amplitude and duration,  S wave amplitude and duration, STJ, ST1, ST2, ST3, T wave amplitude,  QTc, QRS duration, VAT |

Abbreviations: QTc, corrected QT interval; STJ, amplitude at ST junction; ST1, amplitude at the time one tenth of the QT interval has elapsed from ST junction; ST2, amplitude at the time two tenths of the QT interval has elapsed from ST junction; ST3, amplitude at the time three tenths of the QT interval has elapsed from ST junction; VAT, ventricular activation time.

**Supplementary Table 2. Patient characteristics in the external test dataset at the time of ECG and echocardiogram acquisition.**

|  | External Test Dataset (n=47,353) |
| --- | --- |
| Age (years) | 69.8 (13.9) |
| Male, n (%) | 29,805 (62.9) |
| Body Height (cm) | 161.8 (14.0) |
| Body Weight (kg) | 61.1 (14.8) |
| LVEF (%) | 61.1 (13.2) |
| Reduced LVEF, n (%) | 4,187 (8.8) |
| HR (min^-1^) | 74.0 (17.7) |
| PR interval (ms) | 173.8 (49.4) |
| QRS duration (ms) | 108.7 (22.3) |
| QT interval (ms) | 401.9 (44.8) |
| QTc | 438.9 (35.3) |
| QRS axis | 26.0 (42.9) |
| P axis | 38.2 (36.2) |

Data are presented as n (%) or mean (standard deviation).

Abbreviations: LVEF, left ventricular ejection fraction; HR, heart rate; QTc, corrected QT interval.

**Supplementary Table 3. Models’ diagnostic performance on the test dataset.**

|  | Accuracy | Sensitivity | Specificity |
| --- | --- | --- | --- |
| RF | 93.3 (92.7-93.9) | 46.5 (42.9-50.2) | 98.7 (98.4-98.9) |
| SVM | 91.3 (90.7-92.0) | 29.6 (26.3-33.0) | 98.4 (98.0-98.7) |
| LR | 93.1 (92.5-93.7) | 47.9 (44.2-51.6) | 98.3 (97.9-98.6) |
| MLP | 93.1 (92.4-93.6) | 47.6 (44.0-51.3) | 98.2 (97.9-98.5) |

Data are presented as percentages (95% confidence intervals).

Abbreviations: RF, random forest model; SVM, support vector machine model; LR, logistic regression model; MLP, multi-layer perceptron model.

**Supplementary Table 4. Patient characteristics in each cluster extracted as the model’s decision criteria on the test dataset.**

|  | Cluster 1 (n=31) | Cluster 2 (n=21) | Cluster 3 (n=48) | Cluster 4 (n=52) | Cluster 5 (n=31) | Cluster 6 (n=9) |
| --- | --- | --- | --- | --- | --- | --- |
| Age (years) | 46.1 (6.8) | 70.6 (14.6) | 43.1 (9.2) | 53.0 (15.9) | 67.5 (14.2) | 46.3 (16.4) |
| Male, n (%) | 29 (93.6) | 19 (90.5) | 26 (54.2) | 45 (86.5) | 29 (93.6) | 9 (100.0) |
| LVEF (%) | 21.9 (20.0) | 38.5 (17.8) | 25.2 (17.6) | 26.3 (15.4) | 36.0 (13.3) | 13.2 (14.6) |
| Reduced LVEF, n (%) | 26 (83.9) | 12 (57.1) | 38 (79.2) | 45 (86.5) | 24 (77.4) | 8 (88.9) |
| Model’s prediction | 0.729 (0.103) | 0.550 (0.043) | 0.685 (0.099) | 0.638 (0.094) | 0.569 (0.039) | 0.609 (0.086) |
| SHAP value | 0.0036 (0.0100) | 0.0026 (0.0073) | 0.0033 (0.0097) | 0.0031 (0.0084) | 0.0027 (0.0081) | 0.0029 (0.0083) |

Data are presented as n (%) or mean (standard deviation).

Abbreviations: LVEF, left ventricular ejection fraction; SHAP, Shapley Additive Explanations.

**Supplementary Table 5. Patient characteristics in each cluster extracted as the model’s decision criteria on the external test dataset.**

|  | Cluster 1 (n=144) | Cluster 2 (n=314) | Cluster 3 (n=237) | Cluster 4 (n=70) | Cluster 5 (n=54) | Cluster 6 (n=167) | Cluster 7 (n=258) |
| --- | --- | --- | --- | --- | --- | --- | --- |
| Age (years) | 62.1 (11.0) | 76.0 (9.3) | 73.1 (10.7) | 56.3 (8.8) | 69.8 (12.3) | 66.7 (12.8) | 69.3 (14.4) |
| Male, n (%) | 128 (88.9) | 247 (78.7) | 200 (84.4) | 65 (92.9) | 40 (74.1) | 145 (86.8) | 191 (74.0) |
| LVEF (%) | 40.7 (16.1) | 35.0 (14.5) | 38.6 (14.7) | 28.6 (11.8) | 36.9 (16.3) | 37.0 (14.2) | 34.8 (16.6) |
| Reduced LVEF, n (%) | 69 (47.9) | 204 (65.0) | 147 (62.0) | 58 (82.9) | 32 (59.3) | 108 (64.7) | 179 (69.4) |
| Model’s prediction | 0.624 (0.087) | 0.562 (0.055) | 0.566 (0.059) | 0.642 (0.102) | 0.547 (0.041) | 0.543 (0.043) | 0.587 (0.063) |
| SHAP value | 0.0030 (0.0093) | 0.0026 (0.0081) | 0.0027 (0.0088) | 0.0031 (0.0094) | 0.0026 (0.0075) | 0.0025 (0.0073) | 0.0028 (0.0074) |

Data are presented as n (%) or mean (standard deviation).

Abbreviations: LVEF, left ventricular ejection fraction; SHAP, Shapley Additive Explanations.

**Supplementary Table 6. Decision factors and decision criteria in each cluster on the external test dataset.**

|  | Decision Factors | Decision Criteria |
| --- | --- | --- |
| Cluster 1 | R wave amp. in II/V4-6 leads  T wave amp. in I/aVR leads  Q wave dur. In V3-5 leads  R wave dur. in V4 lead | Low voltage in II/V4-6 leads  Negative T wave inversion in I lead  Q wave in V3-5 leads |
| Cluster 2 | T wave amp. in I/II/aVR/V5-6 leads  R wave amp. in Ⅱ/V4 leads  QTc in V2-3 leads  S wave dur. in V2-3 leads | Negative T wave inversion in I/II/V5-6 leads  Low voltage in Ⅱ/V4 leads  QTc prolongation in V2-3 leads  S wave prolongation in V2-3 leads |
| Cluster 3 | R wave amp. in II/V4-5 leads  T wave amp. in Ⅰ/aVR/V5-6 leads  Q wave dur. in V3-4 leads  Q wave amp. in V3 lead  R wave dur. in V3-4 leads | Low voltage in II/V4-5 leads  Negative T wave inversion in Ⅰ/V5-6 leads  Q wave in V3-4 leads |
| Cluster 4 | T wave amp. in I/Ⅱ/aVR/V5-6 leads  R wave amp. in Ⅱ/V3-4 leads  Age  S wave dur. in V2-3 leads | Negative T wave inversion in I/II/V5-6 leads  Low voltage in Ⅱ/V3-4 leads  S wave prolongation in V2-3 leads |
| Cluster 5 | QTc in 12 leads and Ⅲ/aVL/V1-6 leads  R wave amp. in Ⅱ lead | QTc prolongation  Low voltage in II lead |
| Cluster 6 | T wave amp. in I/II/aVR/V5-6 leads  QTc in 12 leads and Ⅲ/V2-4 leads  S wave dur. in V2-3 leads  R wave dur. in V5 lead | Negative T wave inversion in I/II/V5-6 leads  QTc prolongation  S wave prolongation in V2-3 leads  R wave prolongation in V5 lead |
| Cluster 7 | T wave amp. in I/II/aVR/V5-6 leads  QTc in 12 leads and Ⅲ/aVL/V1-6 leads  VAT in V6 lead  R wave amp. in V4 lead  S wave dur. in V2-3 leads | Negative T wave inversion in I/II/V5-6 leads  QTc prolongation  VAT prolongation in V6 lead  Low voltage in V4 lead  S wave prolongation in V2-3 leads |

Decision factors are defined as those ECG parameters that influenced the model’s decision that the ECG was a case of reduced left ventricular ejection fraction. Decision criteria are ECG findings that contributed to the model’s decision, which are interpreted from the relationship between decision factors and SHAP values.

Abbreviations: amp., amplitude; dur., duration; VAT, ventricular activation time; QTc, corrected QT interval.

**Supplementary Table 7. Combinations of six categorized ECG findings in each cluster on the external test dataset**

|  | ECG findings |
| --- | --- |
| Cluster 1 | Low voltage in I/II/V4-6 leads  Negative T wave inversion in I/V5-6 leads  Q wave in V3-6 leads |
| Cluster 2 | Negative T wave inversion in I/V5-6 leads  Low voltage in I/II/V4-6 leads  QTc prolongation  S wave prolongation in V2-3 leads |
| Cluster 3 | Low voltage in I/II/V4-6 leads  Negative T wave inversion in I/V5-6 leads  Q wave in V3-6 leads |
| Cluster 4 | Negative T wave inversion in I/V5-6 leads  Low voltage in I/II/V4-6 leads  S wave prolongation in V2-3 leads |
| Cluster 5 | QTc prolongation  Low voltage in I/II/V4-6 leads |
| Cluster 6 | Negative T wave inversion in I/V5-6 leads  QTc prolongation |
| Cluster 7 | Negative T wave inversion in I/V5-6 leads  QTc prolongation  VAT prolongation in I/V5-6 leads  Low voltage in I/II/V4-6 leads  S wave prolongation in V2-3 leads |

The decision criteria extracted for each cluster could be decomposed into six ECG findings. Data are presented on the combinations of the six ECG findings in each cluster.

Abbreviations: VAT, ventricular activation time; QTc, corrected QT interval.

**Supplementary Fig. 1. Summary plots of SHAP values for the test dataset in the random forest model.**

**
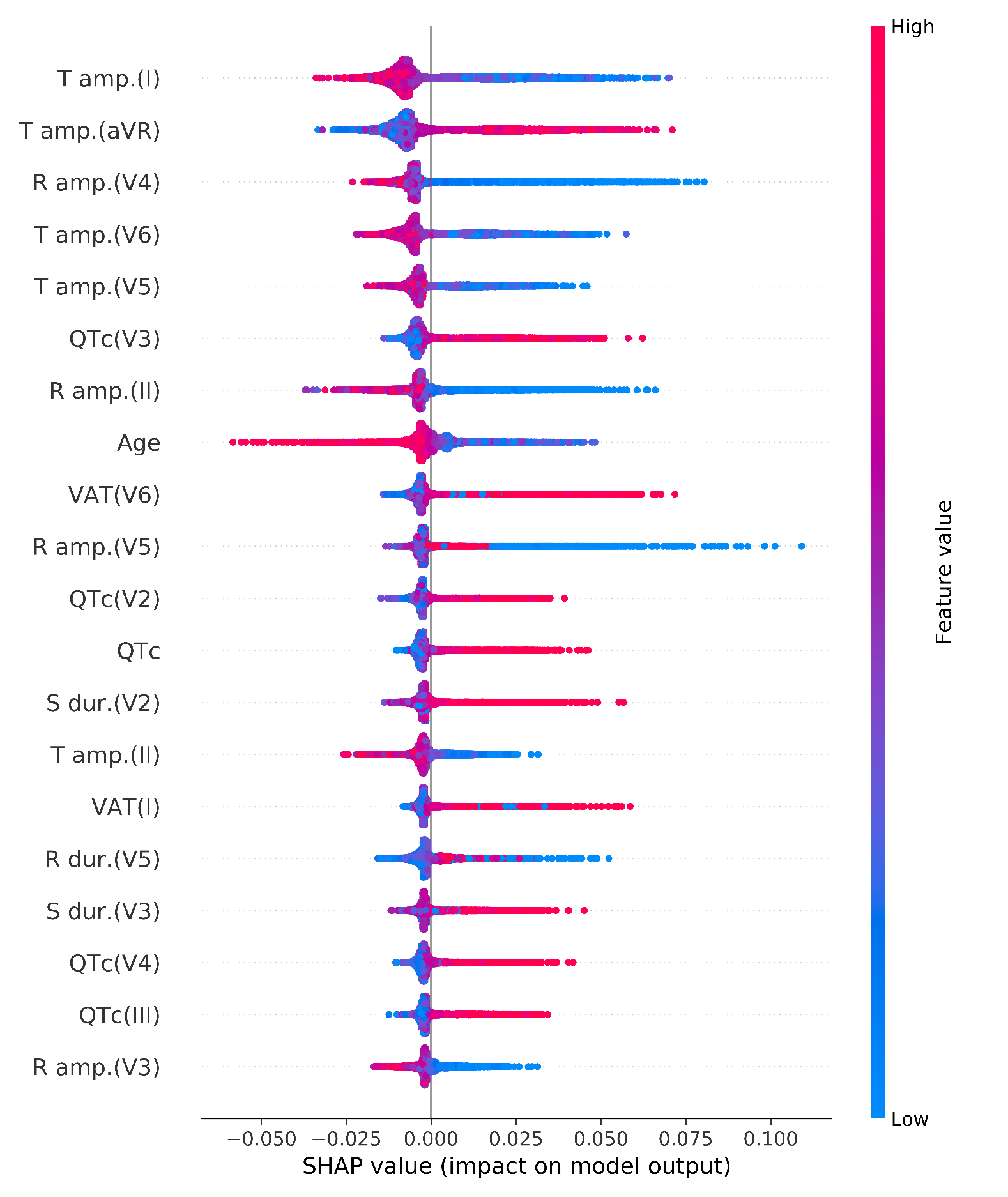
**

The 20 parameters are shown from top to bottom in order of the average of the absolute SHAP values for the test dataset in the random forest model. In other words, the 20 parameters with the highest contribution to the model's prediction are shown. The SHAP value is plotted for each parameter. Each data point approaches red if the feature value is high, and approaches blue if the feature value is low.

Abbreviations: amp., amplitude; QTc, corrected QT interval; VAT, ventricular activation time; dur., duration.

**Supplementary Fig. 2. Summary plots of SHAP values for the test dataset in the support vector machine model.**


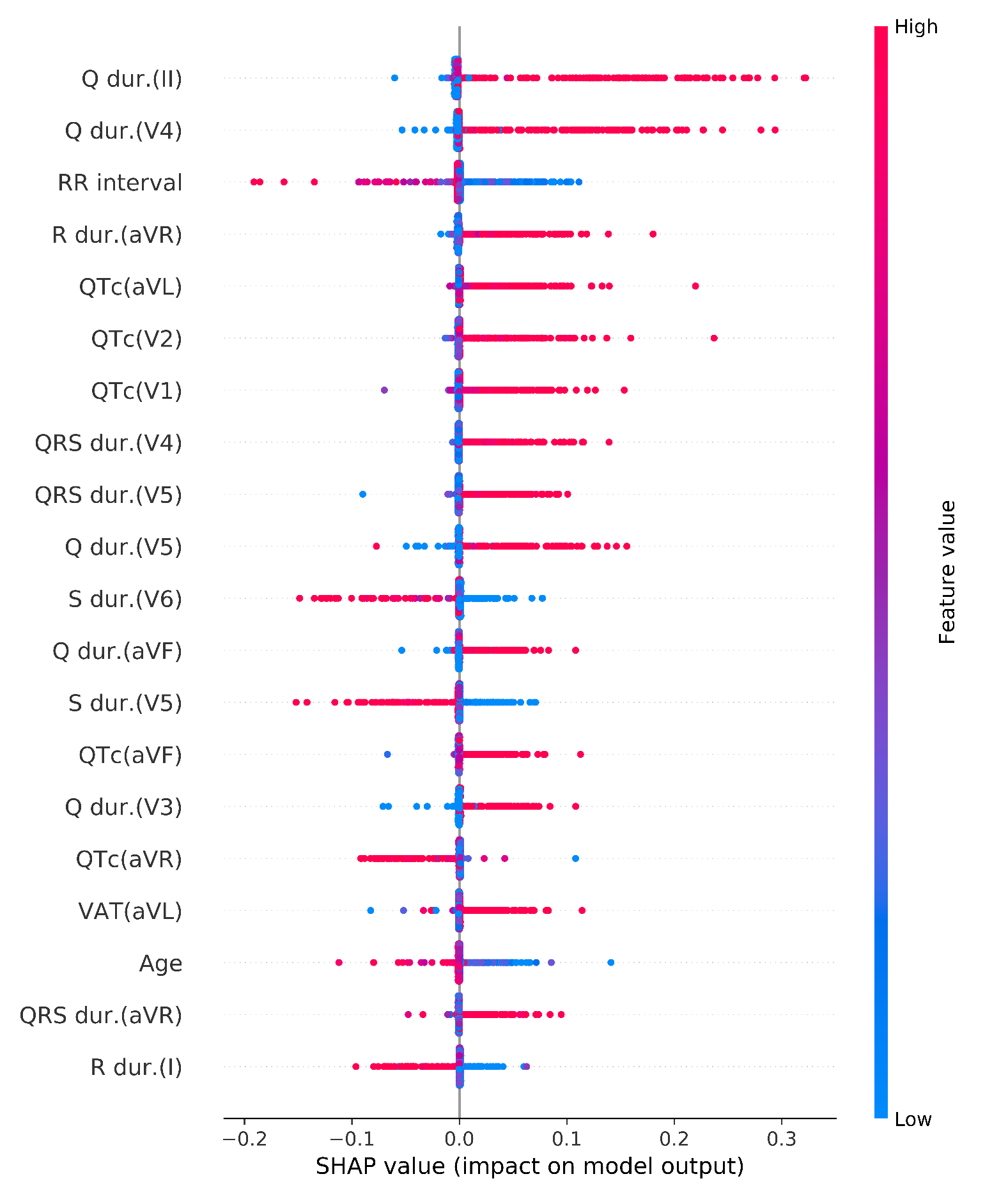


The 20 parameters are shown from top to bottom in order of the average of the absolute SHAP values for the test dataset in the support vector machine model. In other words, the 20 parameters with the highest contribution to the model's prediction are shown. The SHAP value is plotted for each parameter. Each data point approaches red if the feature value is high, and approaches blue if the feature value is low.

Abbreviations: dur., duration; QTc, corrected QT interval; VAT, ventricular activation time.

**Supplementary Fig. 3. Summary plots of SHAP values for the test dataset in the logistic regression model.**


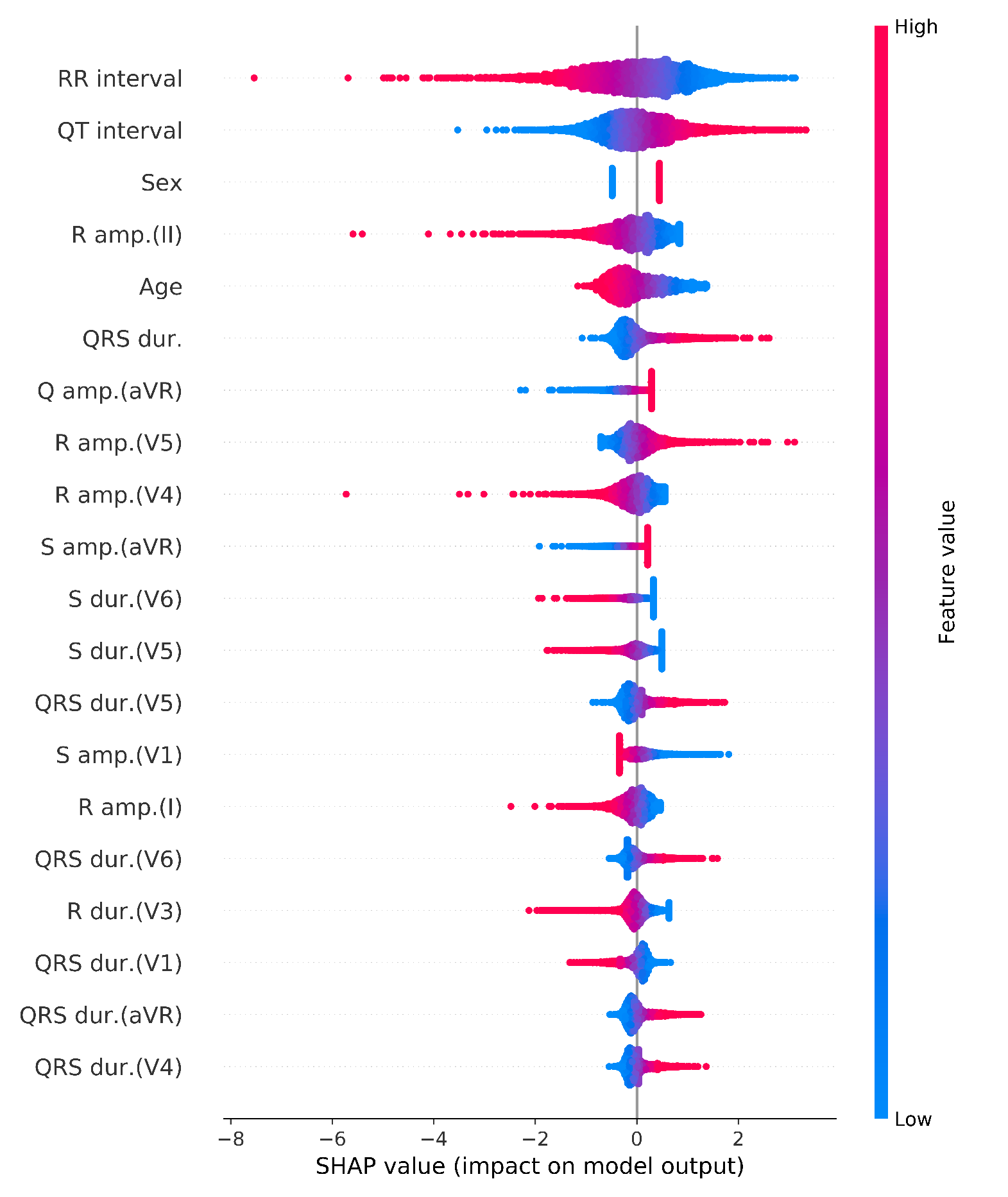


The 20 parameters are shown from top to bottom in order of the average of the absolute SHAP values for the test dataset in the logistic regression model. In other words, the 20 parameters with the highest contribution to the model's prediction are shown. The SHAP value is plotted for each parameter. Each data point approaches red if the feature value is high, and approaches blue if the feature value is low.

Abbreviations: amp., amplitude; dur., duration.

**Supplementary Fig. 4. Summary plots of SHAP values for the test dataset in the multi-layer perceptron model.**


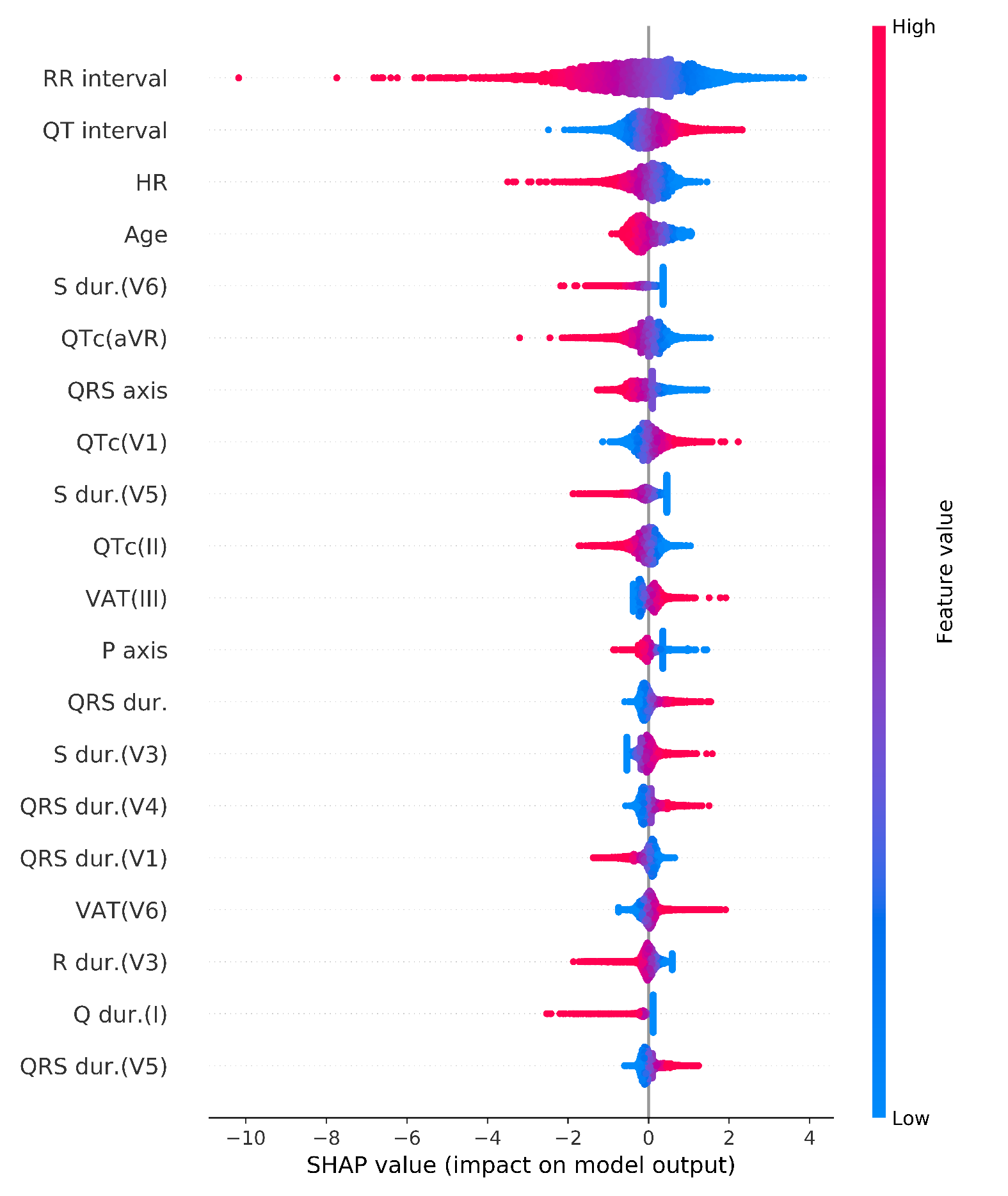


The 20 parameters are shown from top to bottom in order of the average of the absolute SHAP values for the test dataset in the multi-layer perceptron model. In other words, the 20 parameters with the highest contribution to the model's prediction are shown. The SHAP value is plotted for each parameter. Each data point approaches red if the feature value is high, and approaches blue if the feature value is low.

Abbreviations: dur., duration; QTc, corrected QT interval; VAT, ventricular activation time.

**Supplementary Fig. 5. Boxplots of SHAP values in cluster 1 on the test dataset.**

**
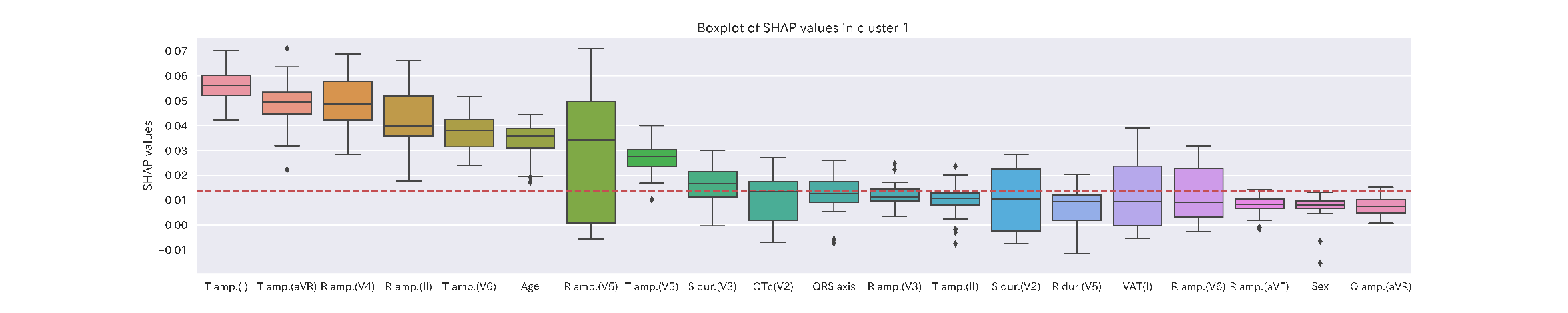
**

In the data included in cluster 1 on the test dataset, boxplots of 20 ECG parameters are shown in order of the median SHAP value from left to right. The red dotted line shows the mean + standard deviation of the SHAP values for all ECG parameters included in the cluster. If the median value of each ECG parameter was above the red dotted line, we defined that ECG parameter as a decision factor, i.e., a factor that influenced the model’s decision that the ECG was a case of reduced LVEF.

Abbreviations: SHAP, Shapley additive explanations; amp., amplitude; dur., duration; VAT, ventricular activation time; QTc, corrected QT interval.

**Supplementary Fig. 6. Boxplots of SHAP values in cluster 2 on the test dataset.**


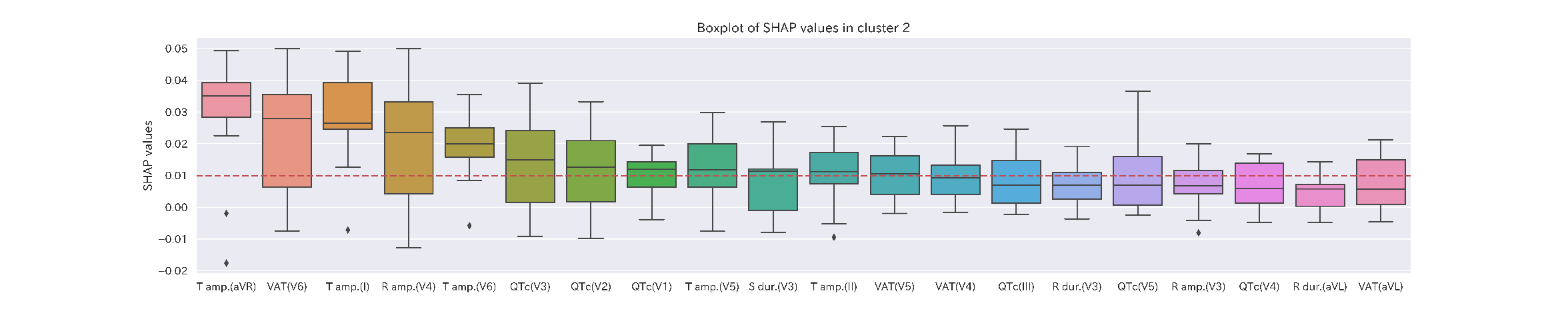


In the data included in cluster 2 on the test dataset, boxplots of 20 ECG parameters are shown in order of the median SHAP value from left to right. The red dotted line shows the mean + standard deviation of the SHAP values for all ECG parameters included in the cluster. If the median value of each ECG parameter was above the red dotted line, we defined that ECG parameter as a decision factor, i.e., a factor that influenced the model’s decision that the ECG was a case of reduced LVEF.

Abbreviations: SHAP, Shapley additive explanations; amp., amplitude; dur., duration; VAT, ventricular activation time; QTc, corrected QT interval.

**Supplementary Fig. 7. Boxplots of SHAP values in cluster 3 on the test dataset.**


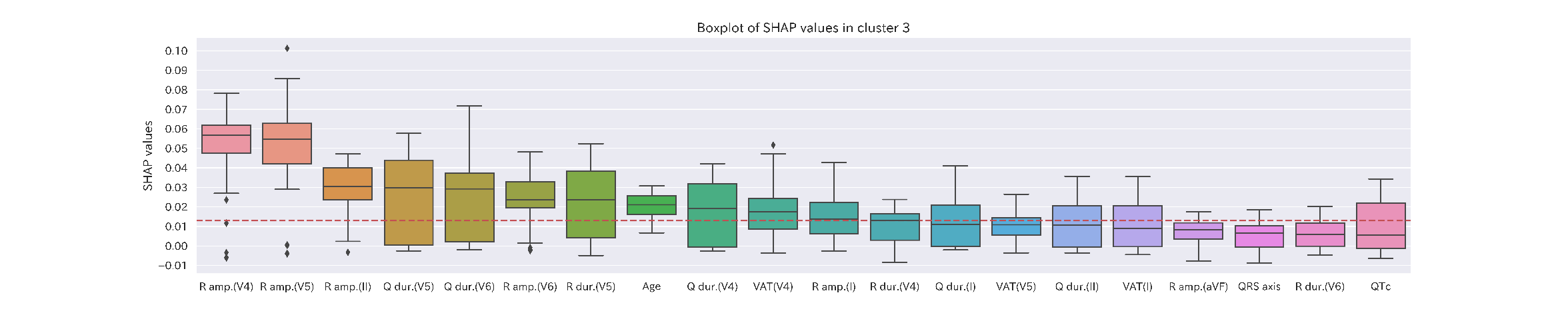


In the data included in cluster 3 on the test dataset, boxplots of 20 ECG parameters are shown in order of the median SHAP value from left to right. The red dotted line shows the mean + standard deviation of the SHAP values for all ECG parameters included in the cluster. If the median value of each ECG parameter was above the red dotted line, we defined that ECG parameter as a decision factor, i.e., a factor that influenced the model’s decision that the ECG was a case of reduced LVEF.

Abbreviations: SHAP, Shapley additive explanations; amp., amplitude; dur., duration; VAT, ventricular activation time; QTc, corrected QT interval.

**Supplementary Fig. 8. Boxplots of SHAP values in cluster 4 on the test dataset.**


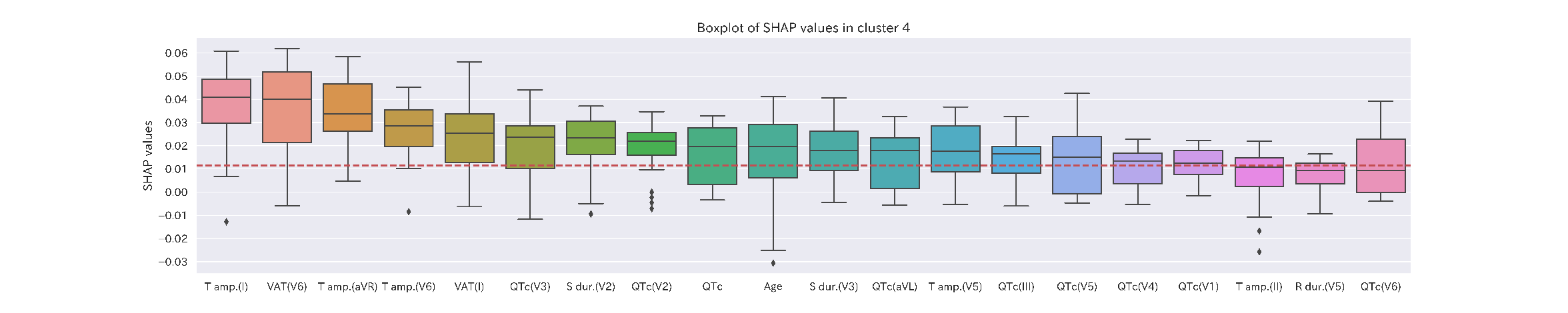


In the data included in cluster 4 on the test dataset, boxplots of 20 ECG parameters are shown in order of the median SHAP value from left to right. The red dotted line shows the mean + standard deviation of the SHAP values for all ECG parameters included in the cluster. If the median value of each ECG parameter was above the red dotted line, we defined that ECG parameter as a decision factor, i.e., a factor that influenced the model’s decision that the ECG was a case of reduced LVEF.

Abbreviations: SHAP, Shapley additive explanations; amp., amplitude; dur., duration; VAT, ventricular activation time; QTc, corrected QT interval.

**Supplementary Fig. 9. Boxplots of SHAP values in cluster 5 on the test dataset.**


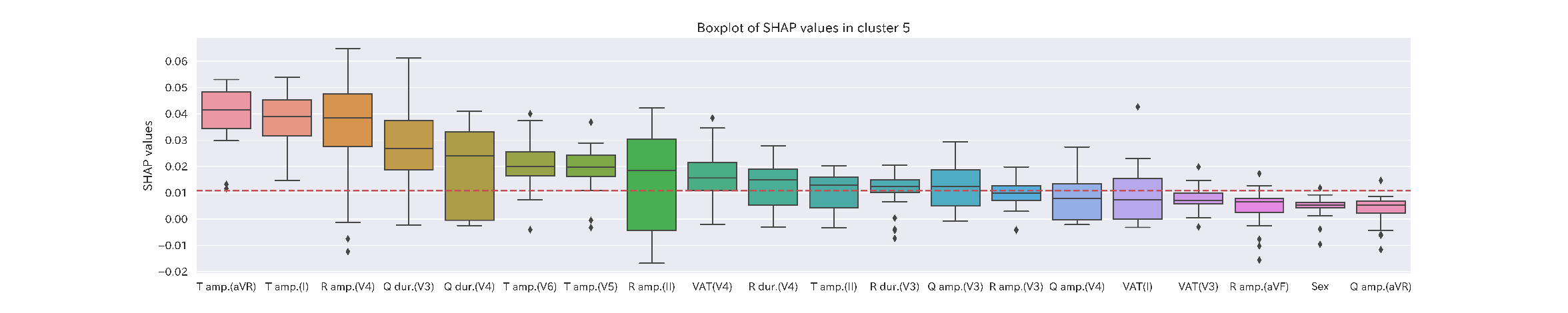


In the data included in cluster 5 on the test dataset, boxplots of 20 ECG parameters are shown in order of the median SHAP value from left to right. The red dotted line shows the mean + standard deviation of the SHAP values for all ECG parameters included in the cluster. If the median value of each ECG parameter was above the red dotted line, we defined that ECG parameter as a decision factor, i.e., a factor that influenced the model’s decision that the ECG was a case of reduced LVEF.

Abbreviations: SHAP, Shapley additive explanations; amp., amplitude; dur., duration; VAT, ventricular activation time; QTc, corrected QT interval.

**Supplementary Fig. 10. Scatter plots of ECG parameters and SHAP values in cluster 1 on the test dataset.**


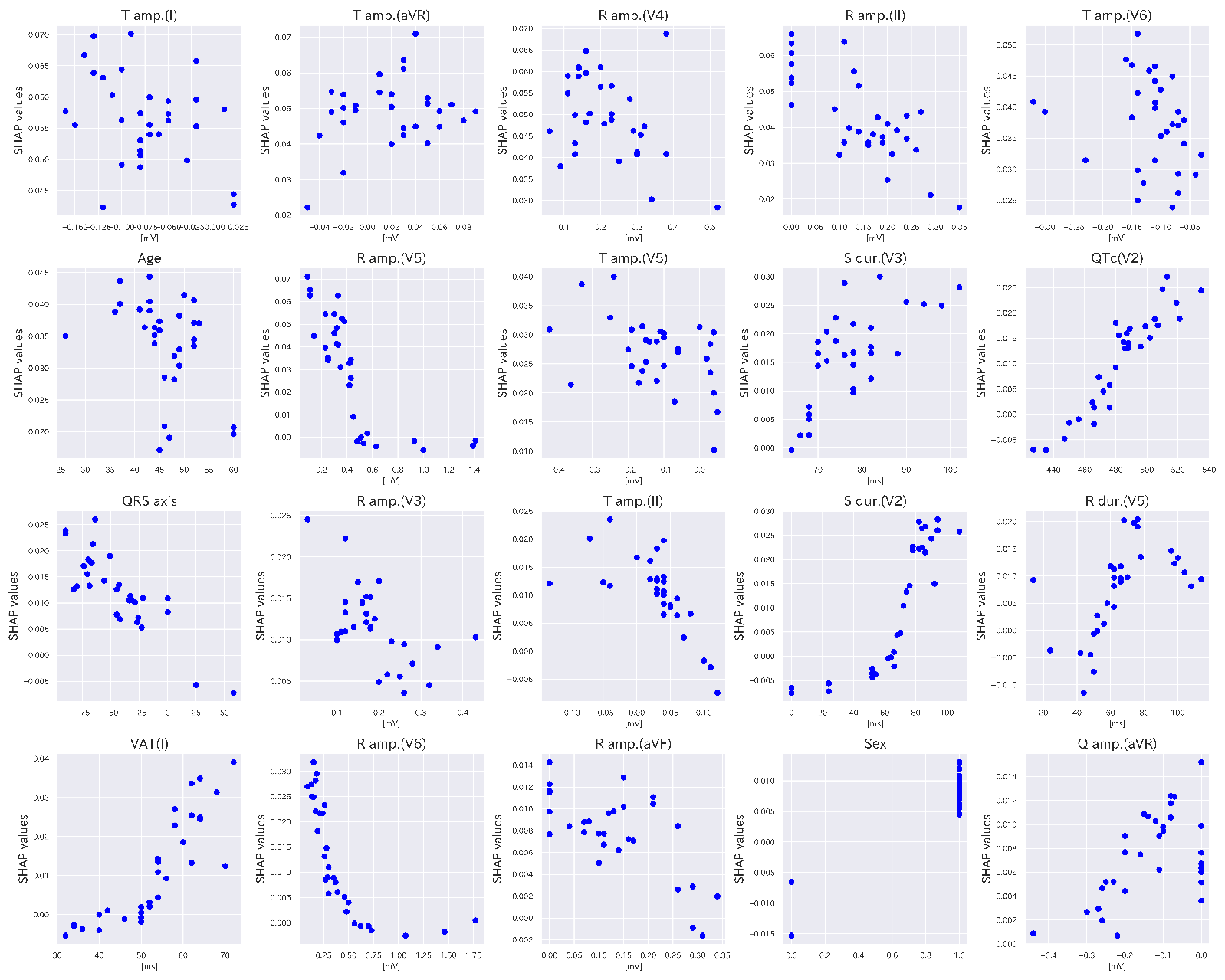


For the data included in cluster 1 on the test dataset, scatter plots of 20 ECG parameters and SHAP values are shown in order of median SHAP value from top left to bottom right.

Abbreviations: SHAP, Shapley additive explanations; amp., amplitude; dur., duration; VAT, ventricular activation time; QTc, corrected QT interval.

**Supplementary Fig. 11. Scatter plots of ECG parameters and SHAP values in cluster 2 on the test dataset.**


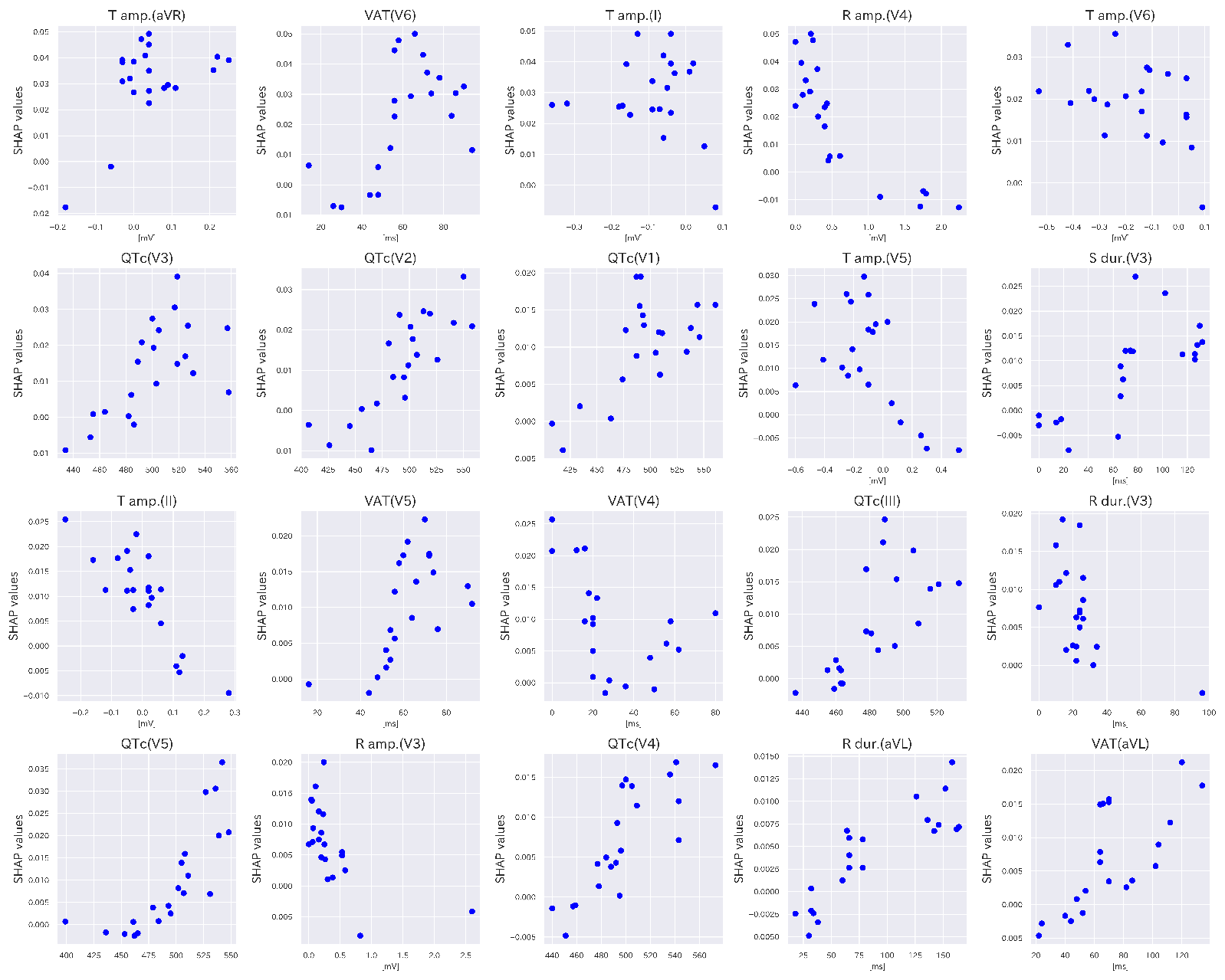


For the data included in cluster 2 on the test dataset, scatter plots of 20 ECG parameters and SHAP values are shown in order of median SHAP value from top left to bottom right.

Abbreviations: SHAP, Shapley additive explanations; amp., amplitude; dur., duration; VAT, ventricular activation time; QTc, corrected QT interval.

**Supplementary Fig. 12. Scatter plots of ECG parameters and SHAP values in cluster 3 on the test dataset.**


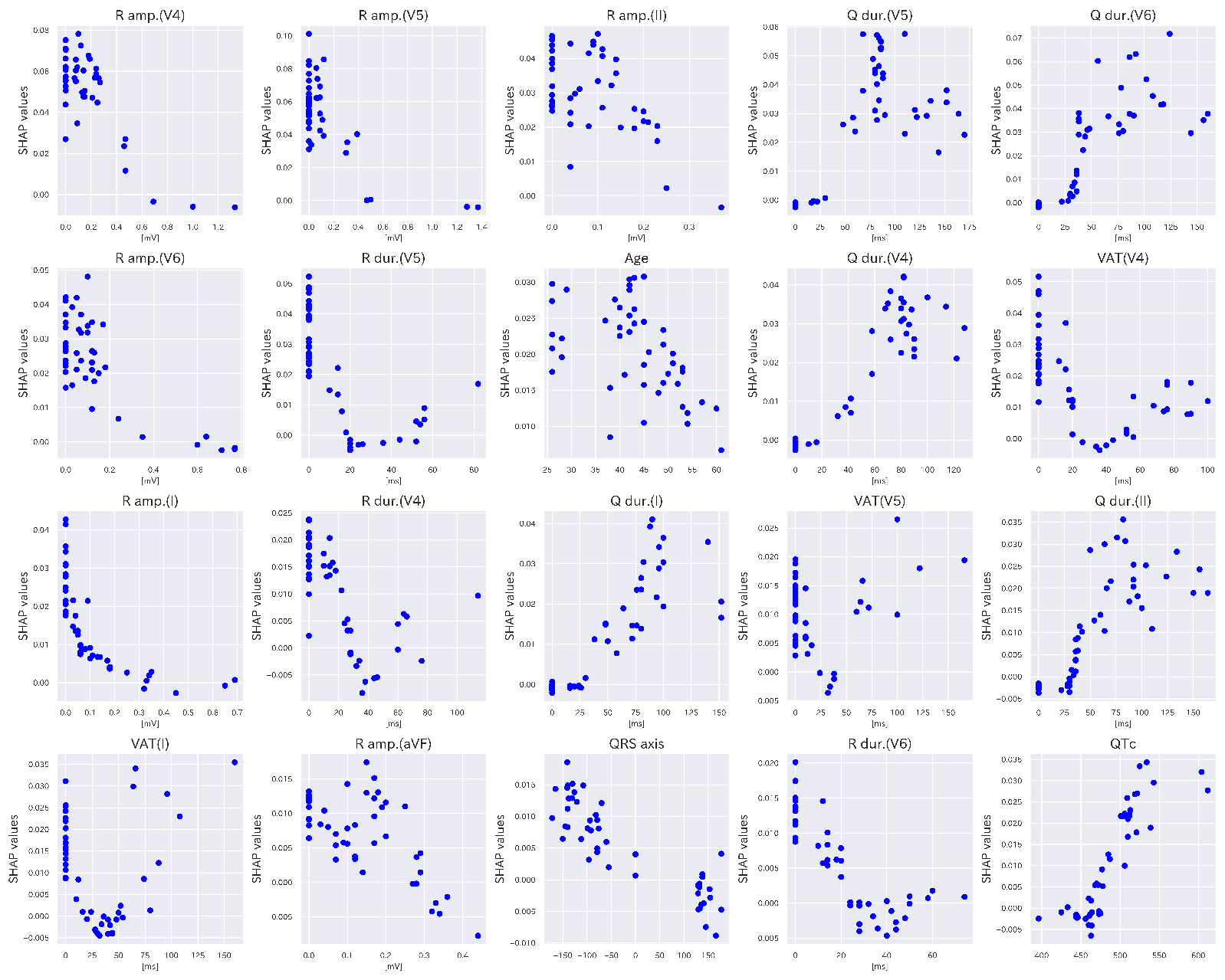


For the data included in cluster 3 on the test dataset, scatter plots of 20 ECG parameters and SHAP values are shown in order of median SHAP value from top left to bottom right.

Abbreviations: SHAP, Shapley additive explanations; amp., amplitude; dur., duration; VAT, ventricular activation time; QTc, corrected QT interval.

**Supplementary Fig. 13. Scatter plots of ECG parameters and SHAP values in cluster 4 on the test dataset.**


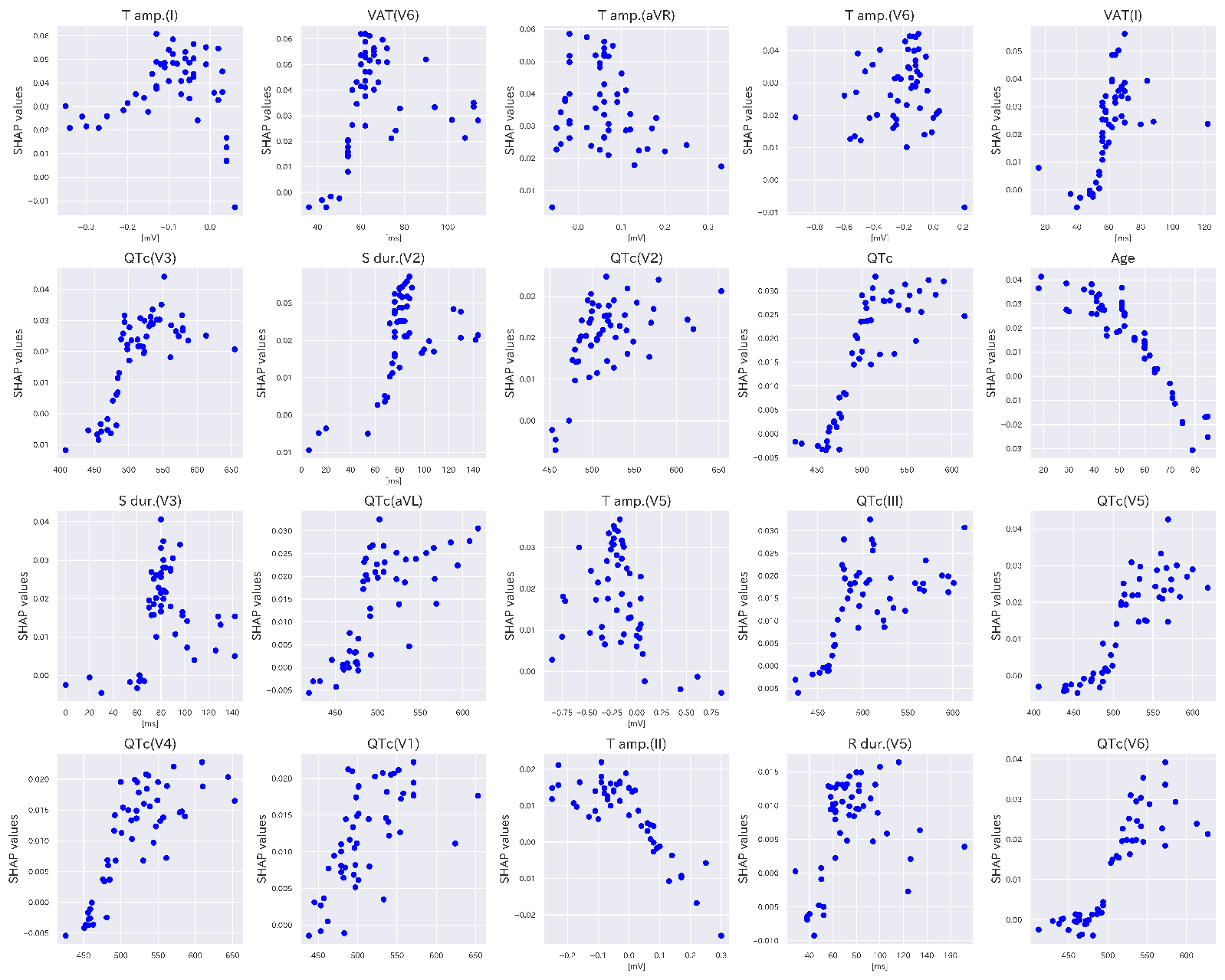


For the data included in cluster 4 on the test dataset, scatter plots of 20 ECG parameters and SHAP values are shown in order of median SHAP value from top left to bottom right.

Abbreviations: SHAP, Shapley additive explanations; amp., amplitude; dur., duration; VAT, ventricular activation time; QTc, corrected QT interval.

**Supplementary Fig. 14. Scatter plots of ECG parameters and SHAP values in cluster 5 on the test dataset.**


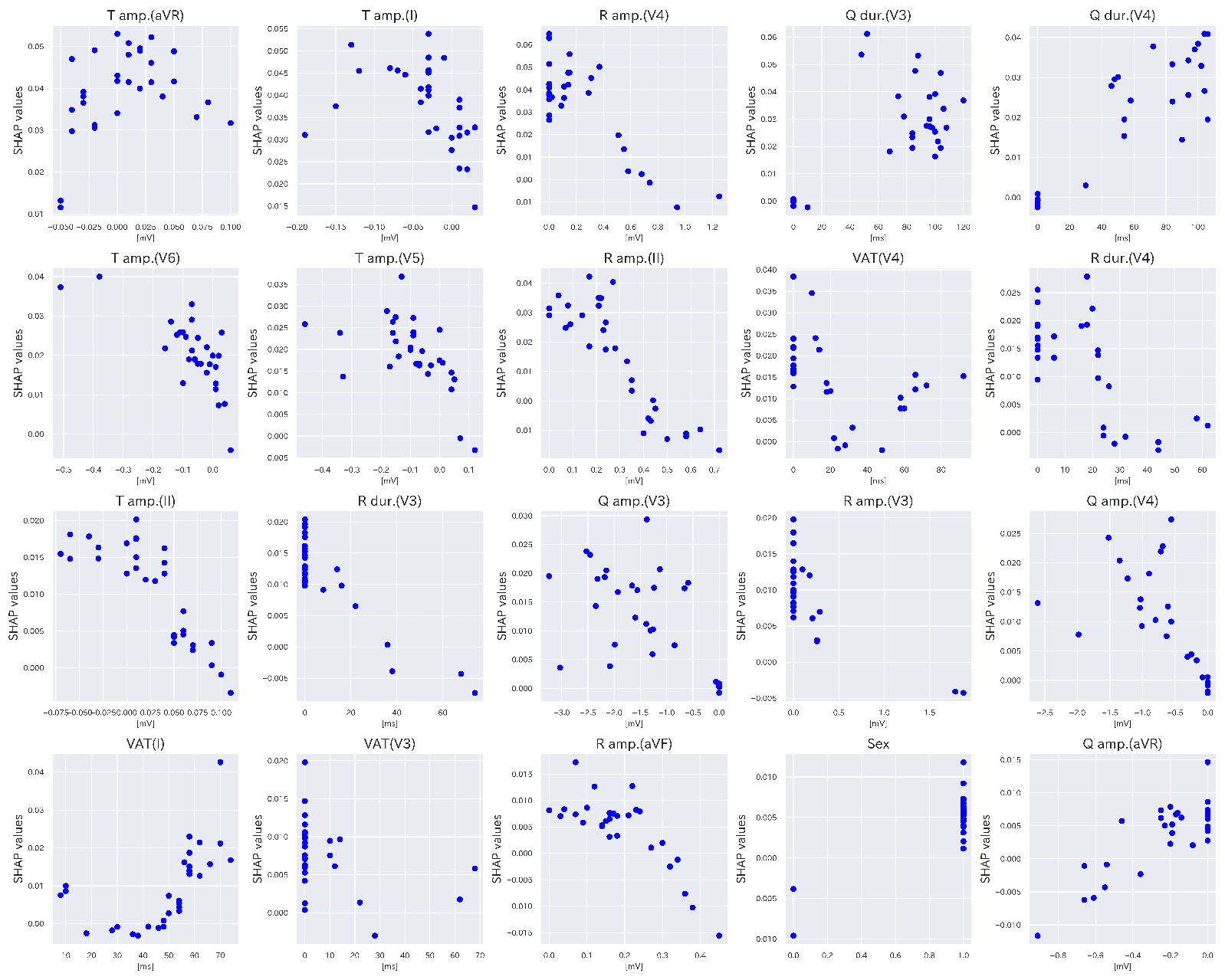


For the data included in cluster 5 on the test dataset, scatter plots of 20 ECG parameters and SHAP values are shown in order of median SHAP value from top left to bottom right.

Abbreviations: SHAP, Shapley additive explanations; amp., amplitude; dur., duration; VAT, ventricular activation time; QTc, corrected QT interval.

**Supplementary Fig. 15. Receiver operating curves of the random forest model applied to the test dataset and the external test dataset.**

**
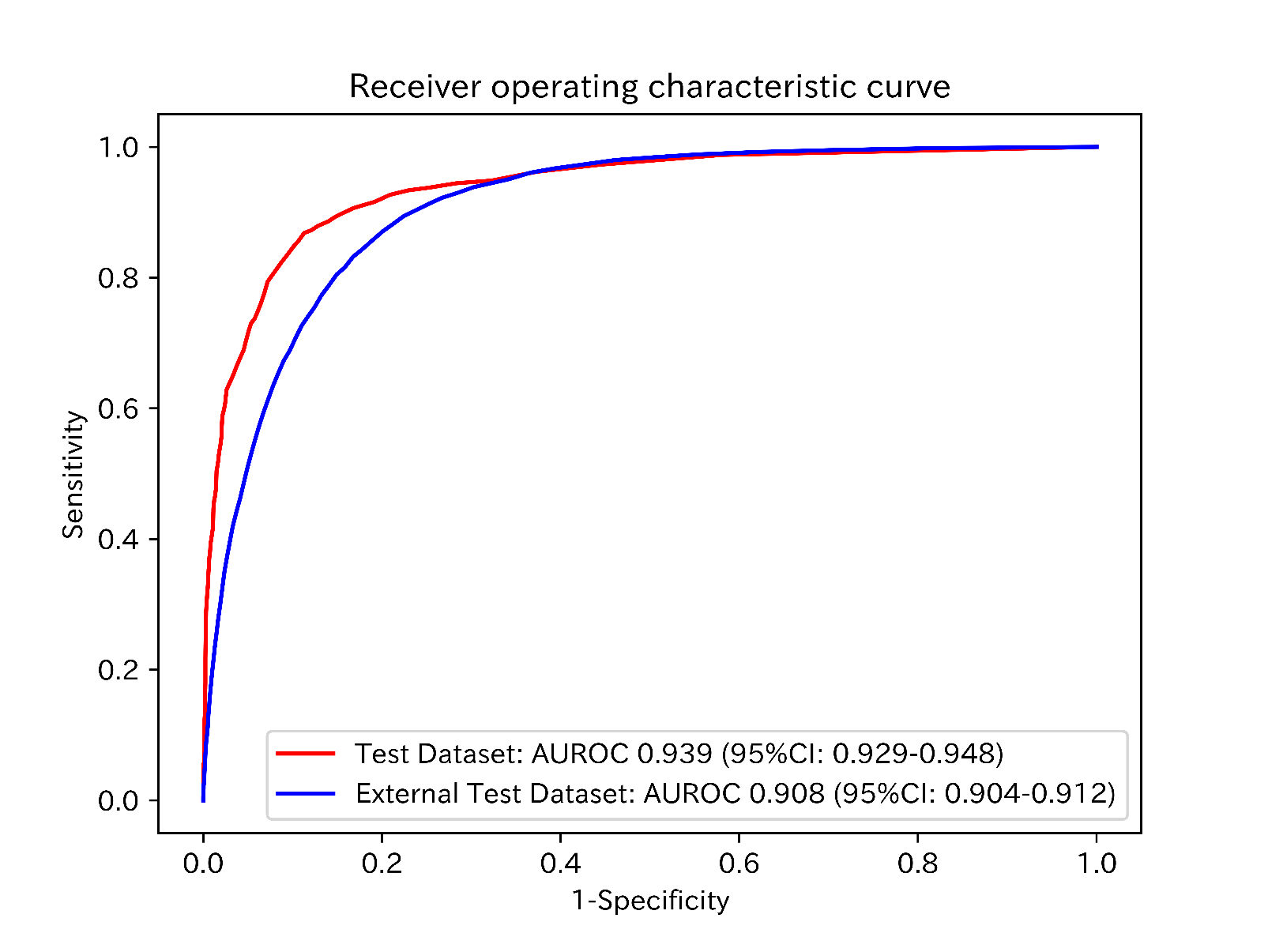
**

Red and blue lines represent the receiver operating characteristic curves of the random forest model applied to the test dataset and the external test dataset, respectively.

Abbreviations: AUROC, area under the receiver operating characteristics curve; CI, confidence interval.

**Supplementary Fig. 16. Visualization of the two-dimensional SHAP values of the external test dataset color mapped by the LVEF values.**

**
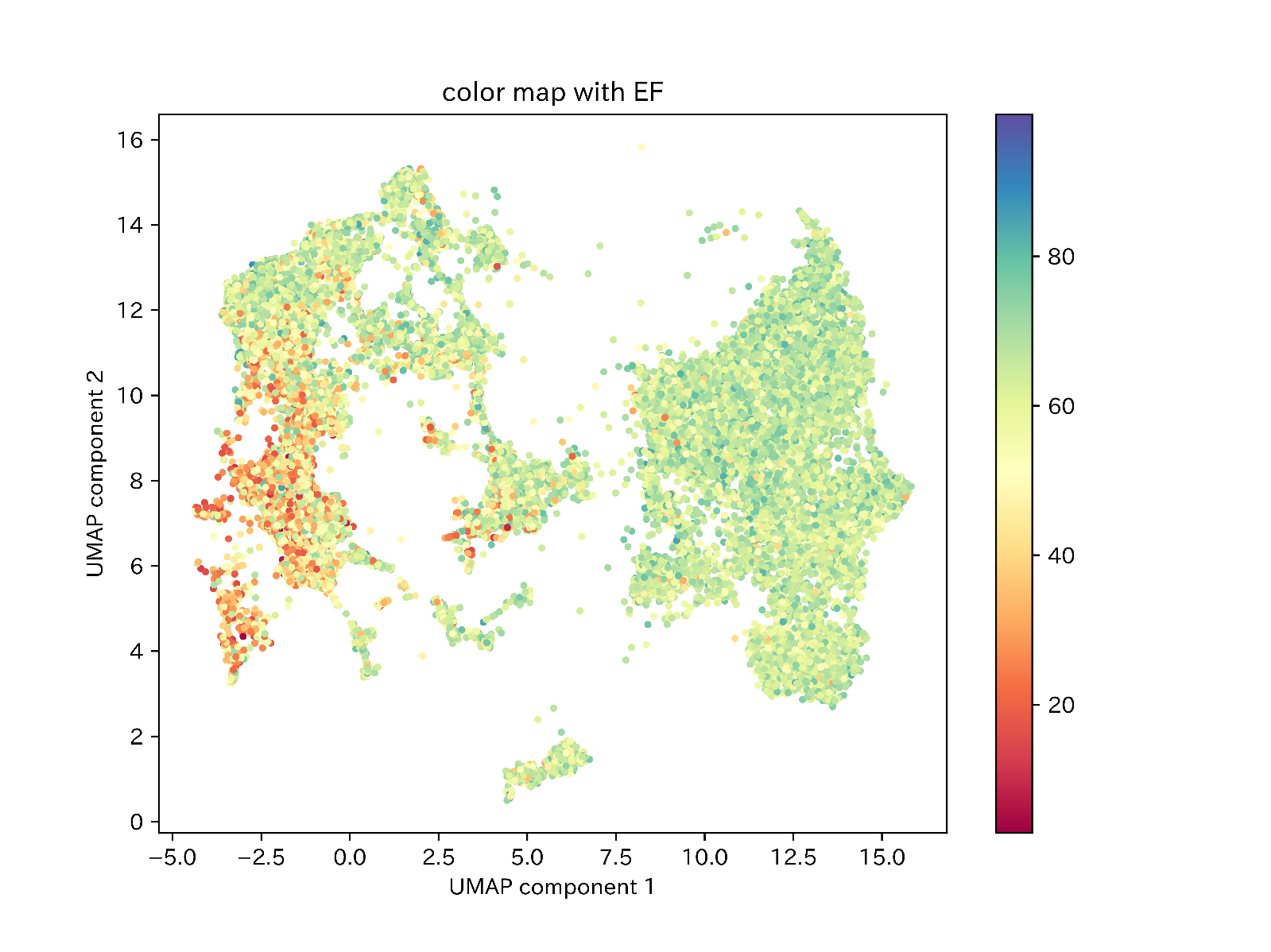
**

Abbreviations: UMAP, uniform manifold approximation and projection.

**Supplementary Fig. 17. Visualization of the two-dimensional SHAP values of the external test dataset color mapped by the model’s predictive values.**


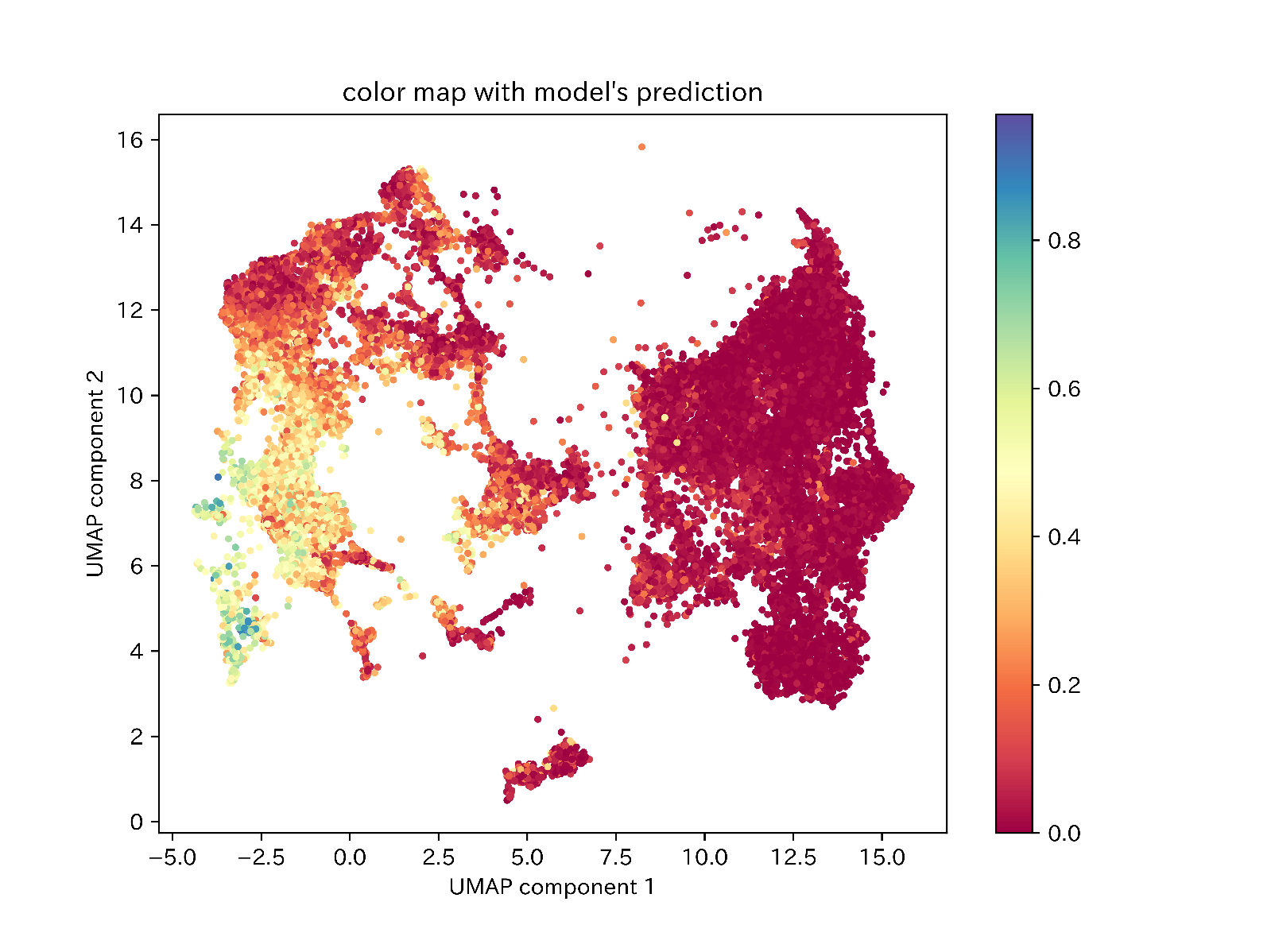


Abbreviations: UMAP, uniform manifold approximation and projection.

**Supplementary Fig. 18. Clustering of two-dimensional SHAP values for ECGs in the external test dataset by which the model predicted reduced LVEF.**


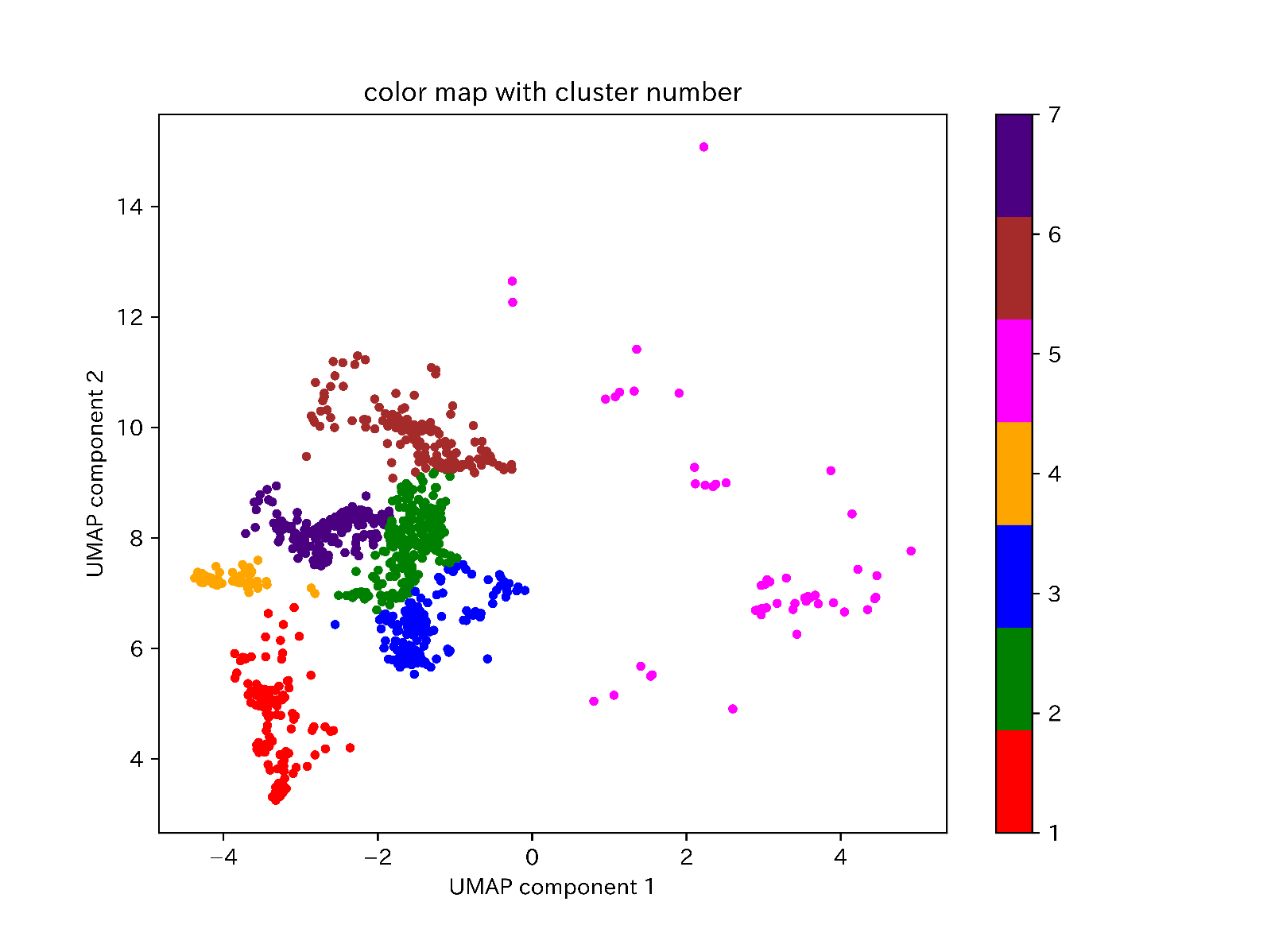


The 1,244 ECGs by which the model predicted reduced LVEF, excluding those with a paced rhythm, were clustered by adapting a Variational Bayesian Gaussian Mixture Model to the two-dimensional SHAP values. The clusters are classified from 1 to 7, and the data points belonging to each cluster are colored red, green, blue, orange, pink, brown, or purple.

Abbreviations: UMAP, uniform manifold approximation and projection.

**Supplementary Fig. 19. Boxplots of SHAP values in cluster 1 on the external test dataset.**


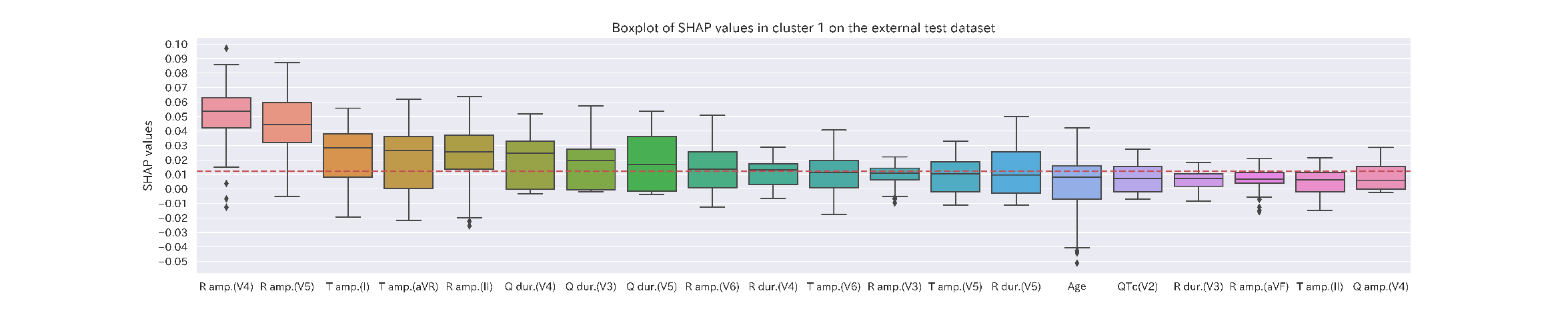


In the data included in cluster 1 on the external test dataset, boxplots of 20 ECG parameters are shown in order of the median SHAP value from left to right. The red dotted line shows the mean + standard deviation of the SHAP values for all ECG parameters included in the cluster. If the median value of each ECG parameter was above the red dotted line, we defined that ECG parameter as a decision factor, i.e., a factor that influenced the model’s decision that the ECG was a case of reduced LVEF.

Abbreviations: SHAP, Shapley additive explanations; amp., amplitude; dur., duration; QTc, corrected QT interval.

**Supplementary Fig. 20. Boxplots of SHAP values in cluster 2 on the external test dataset.**


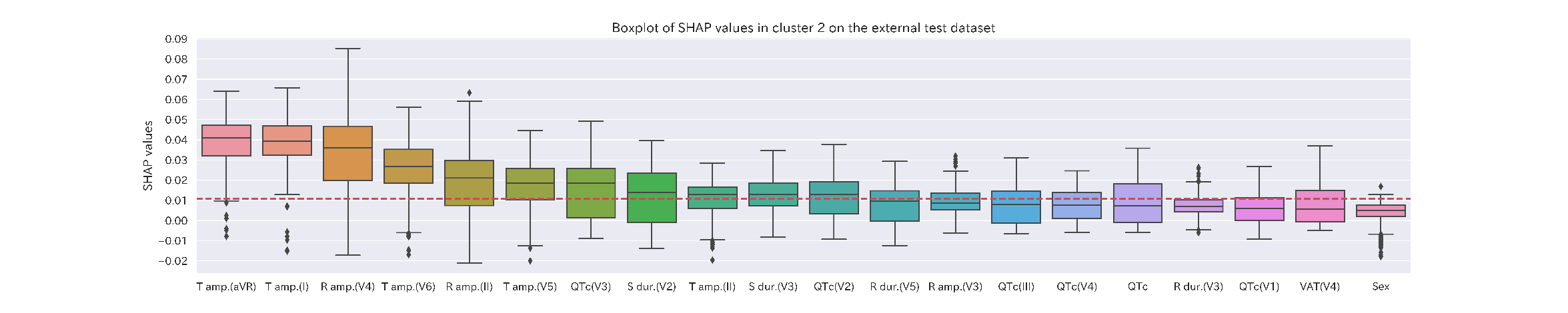


In the data included in cluster 2 on the external test dataset, boxplots of 20 ECG parameters are shown in order of the median SHAP value from left to right. The red dotted line shows the mean + standard deviation of the SHAP values for all ECG parameters included in the cluster. If the median value of each ECG parameter was above the red dotted line, we defined that ECG parameter as a decision factor, i.e., a factor that influenced the model’s decision that the ECG was a case of reduced LVEF.

Abbreviations: SHAP, Shapley additive explanations; amp., amplitude; dur., duration; QTc, corrected QT interval; VAT, ventricular activation time.

**Supplementary Fig. 21. Boxplots of SHAP values in cluster 3 on the external test dataset.**

**
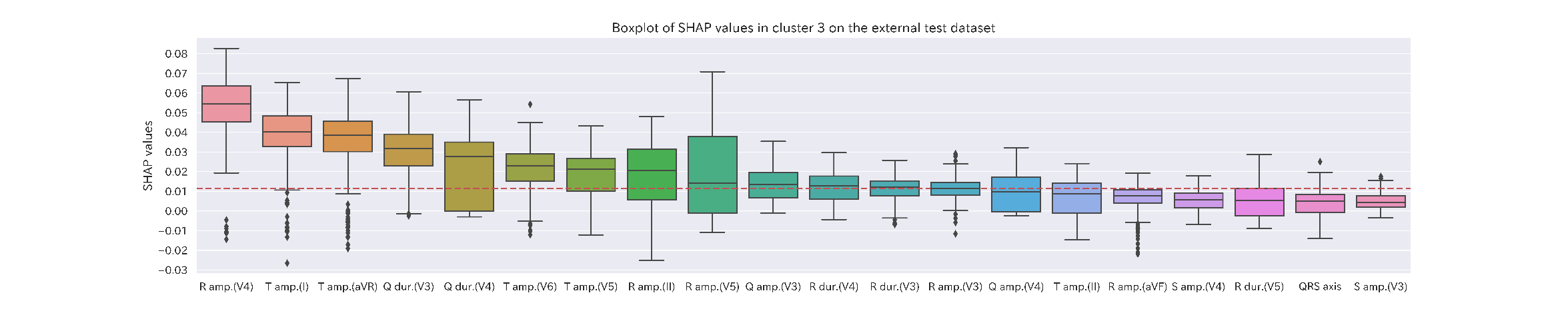
**

In the data included in cluster 3 on the external test dataset, boxplots of 20 ECG parameters are shown in order of the median SHAP value from left to right. The red dotted line shows the mean + standard deviation of the SHAP values for all ECG parameters included in the cluster. If the median value of each ECG parameter was above the red dotted line, we defined that ECG parameter as a decision factor, i.e., a factor that influenced the model’s decision that the ECG was a case of reduced LVEF.

Abbreviations: SHAP, Shapley additive explanations; amp., amplitude; dur., duration.

**Supplementary Fig. 22. Boxplots of SHAP values in cluster 4 on the external test dataset.**

**
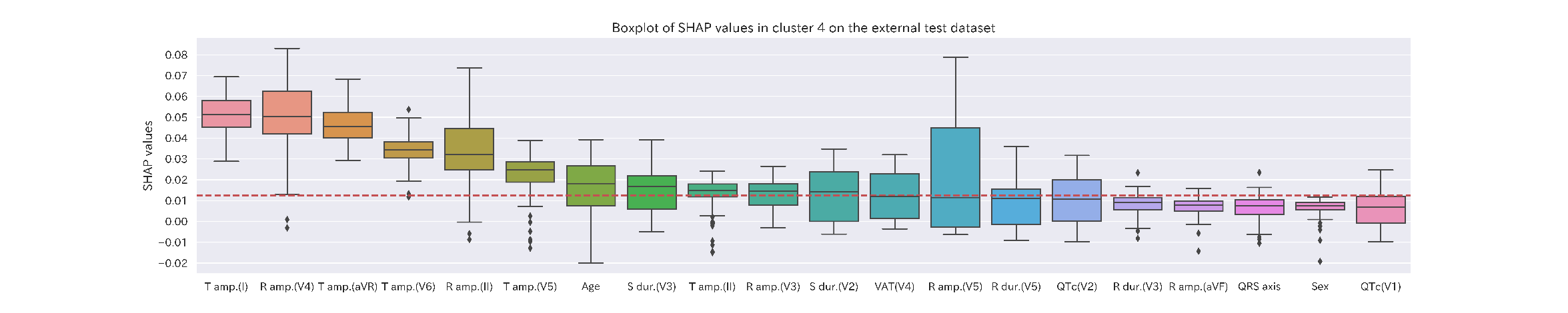
**

In the data included in cluster 4 on the external test dataset, boxplots of 20 ECG parameters are shown in order of the median SHAP value from left to right. The red dotted line shows the mean + standard deviation of the SHAP values for all ECG parameters included in the cluster. If the median value of each ECG parameter was above the red dotted line, we defined that ECG parameter as a decision factor, i.e., a factor that influenced the model’s decision that the ECG was a case of reduced LVEF.

Abbreviations: SHAP, Shapley additive explanations; amp., amplitude; dur., duration; VAT, ventricular activation time; QTc, corrected QT interval.

**Supplementary Fig. 23. Boxplots of SHAP values in cluster 5 on the external test dataset.**


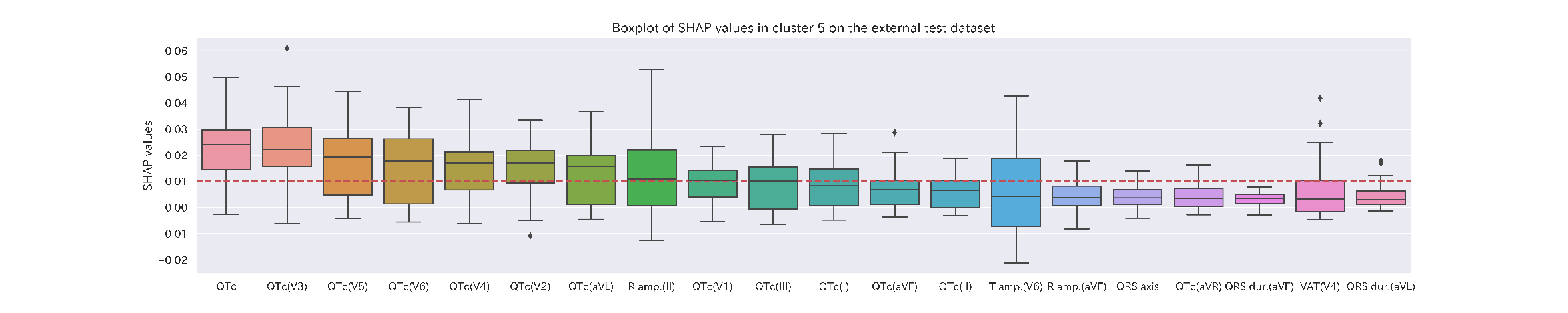


In the data included in cluster 5 on the external test dataset, boxplots of 20 ECG parameters are shown in order of the median SHAP value from left to right. The red dotted line shows the mean + standard deviation of the SHAP values for all ECG parameters included in the cluster. If the median value of each ECG parameter was above the red dotted line, we defined that ECG parameter as a decision factor, i.e., a factor that influenced the model’s decision that the ECG was a case of reduced LVEF.

Abbreviations: SHAP, Shapley additive explanations; amp., amplitude; dur., duration; VAT, ventricular activation time; QTc, corrected QT interval.

**Supplementary Fig. 24. Boxplots of SHAP values in cluster 6 on the external test dataset.**

**
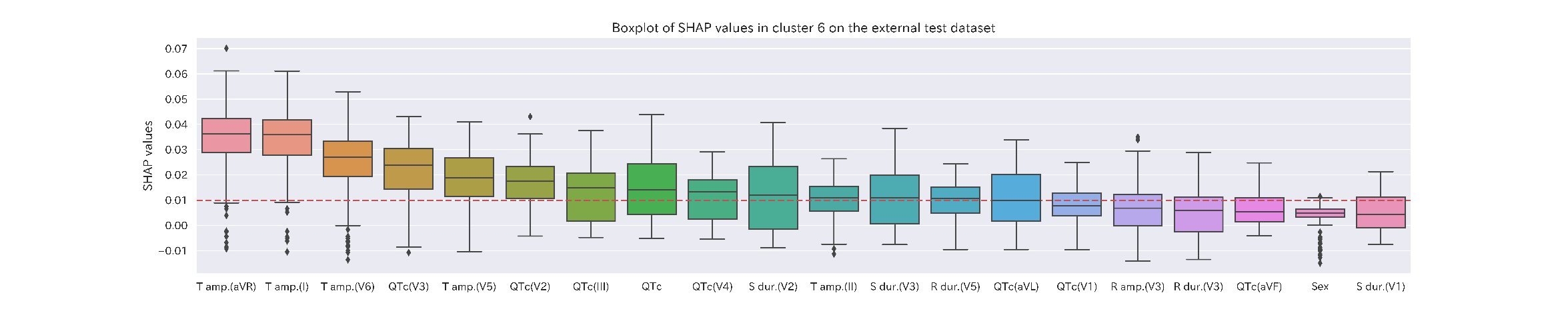
**

In the data included in cluster 6 on the external test dataset, boxplots of 20 ECG parameters are shown in order of the median SHAP value from left to right. The red dotted line shows the mean + standard deviation of the SHAP values for all ECG parameters included in the cluster. If the median value of each ECG parameter was above the red dotted line, we defined that ECG parameter as a decision factor, i.e., a factor that influenced the model’s decision that the ECG was a case of reduced LVEF.

Abbreviations: SHAP, Shapley additive explanations; amp., amplitude; dur., duration; QTc, corrected QT interval.

**Supplementary Fig. 25. Boxplots of SHAP values in cluster 7 on the external test dataset.**


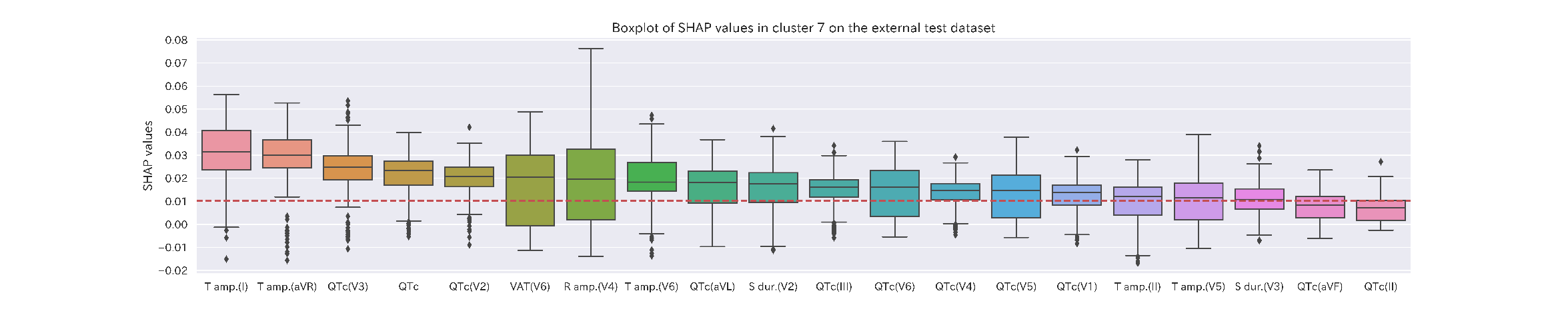


In the data included in cluster 7 on the external test dataset, boxplots of 20 ECG parameters are shown in order of the median SHAP value from left to right. The red dotted line shows the mean + standard deviation of the SHAP values for all ECG parameters included in the cluster. If the median value of each ECG parameter was above the red dotted line, we defined that ECG parameter as a decision factor, i.e., a factor that influenced the model’s decision that the ECG was a case of reduced LVEF.

Abbreviations: SHAP, Shapley additive explanations; amp., amplitude; dur., duration; VAT, ventricular activation time; QTc, corrected QT interval.

**Supplementary Fig. 26. Scatter plots of ECG parameters and SHAP values in cluster 1 on the external test dataset.**

**
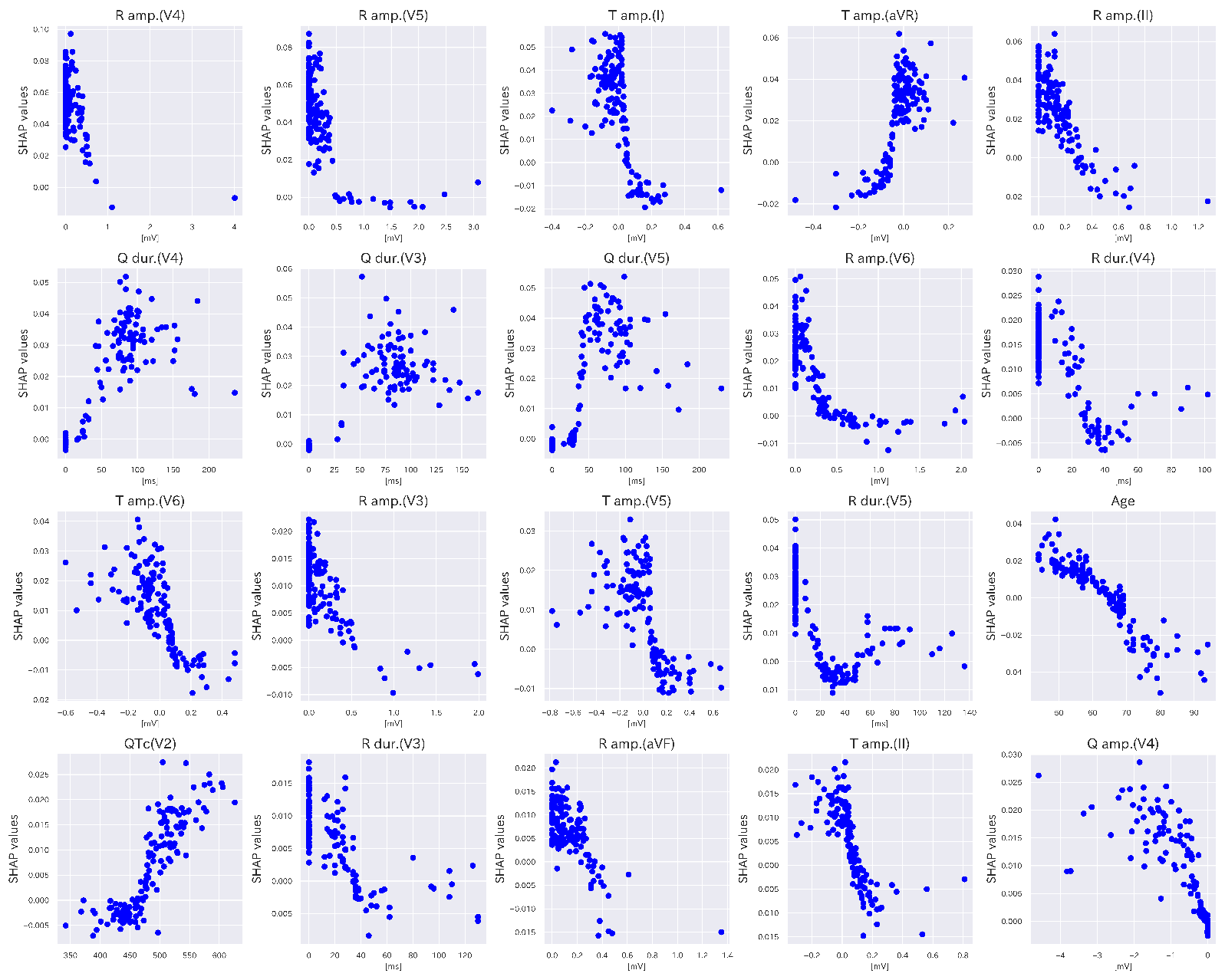
**

For the data included in cluster 1 on the external test dataset, scatter plots of 20 ECG parameters and SHAP values are shown in order of median SHAP value from top left to bottom right.

Abbreviations: SHAP, Shapley additive explanations; amp., amplitude; dur., duration; QTc, corrected QT interval.

**Supplementary Fig. 27. Scatter plots of ECG parameters and SHAP values in cluster 2 on the external test dataset.**

**
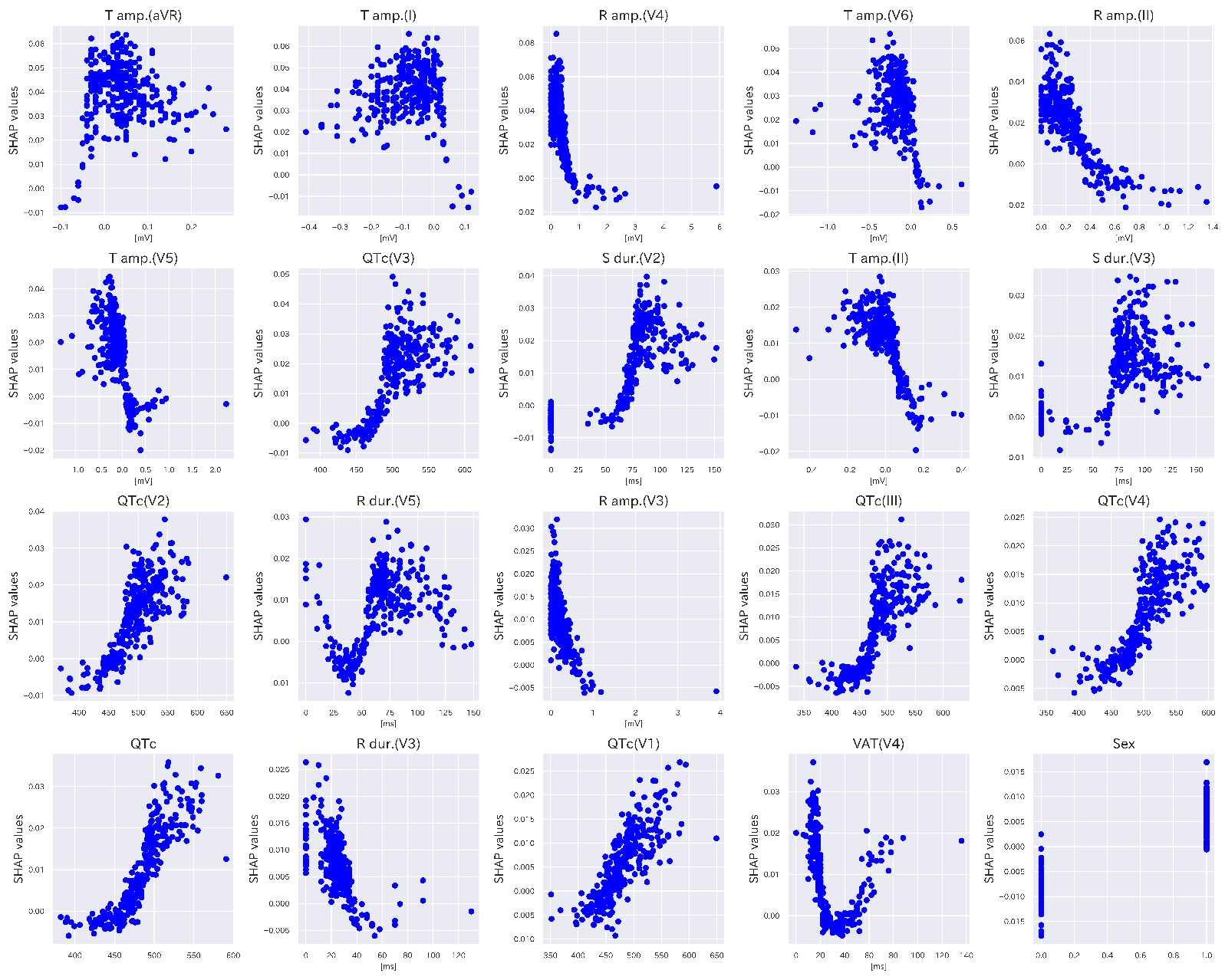
**

For the data included in cluster 2 on the external test dataset, scatter plots of 20 ECG parameters and SHAP values are shown in order of median SHAP value from top left to bottom right.

Abbreviations: SHAP, Shapley additive explanations; amp., amplitude; dur., duration; QTc, corrected QT interval; VAT, ventricular activation time.

**Supplementary Fig. 28. Scatter plots of ECG parameters and SHAP values in cluster 3 on the external test dataset.**

**
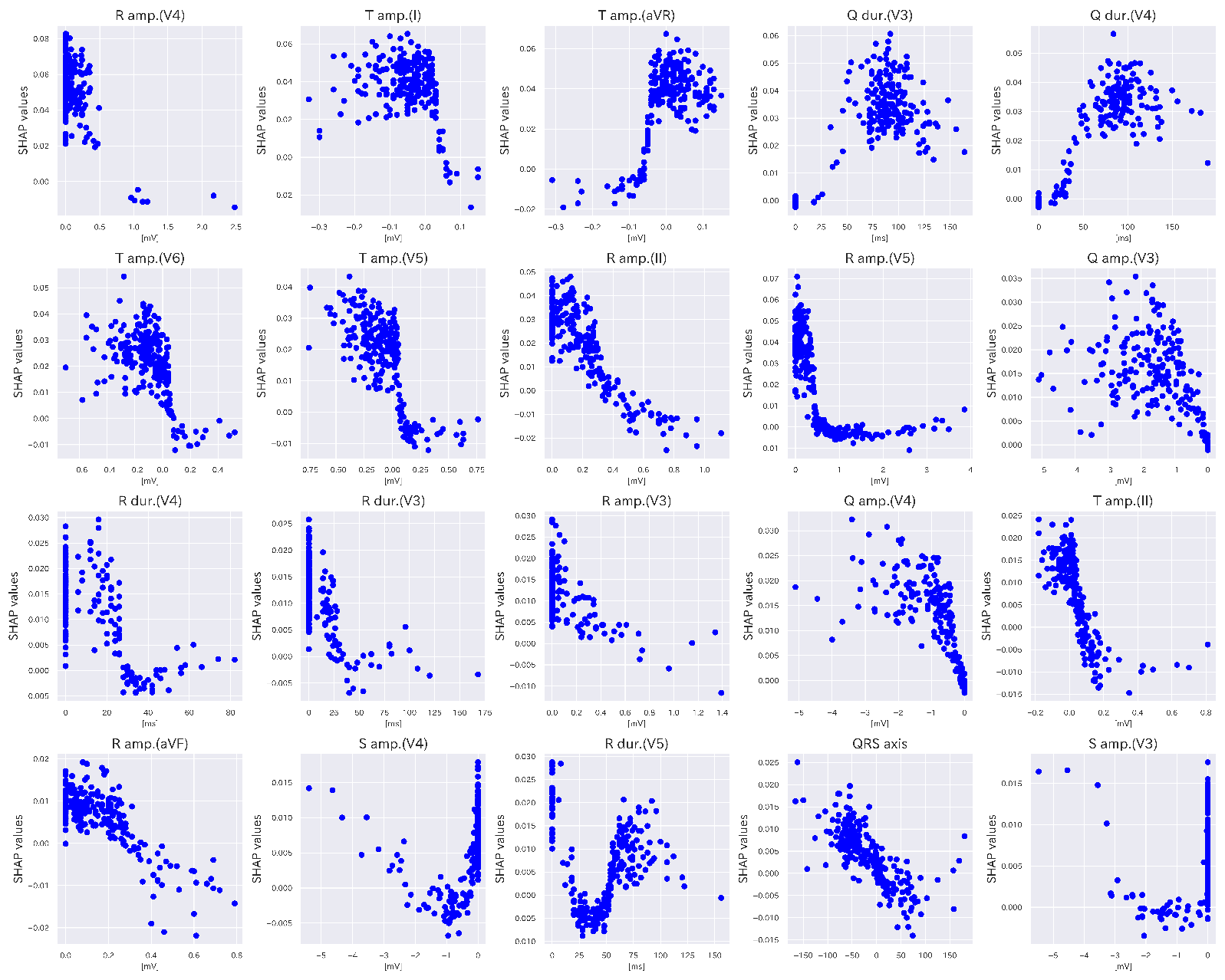
**

For the data included in cluster 3 on the external test dataset, scatter plots of 20 ECG parameters and SHAP values are shown in order of median SHAP value from top left to bottom right.

Abbreviations: SHAP, Shapley additive explanations; amp., amplitude; dur., duration.

**Supplementary Fig. 29. Scatter plots of ECG parameters and SHAP values in cluster 4 on the external test dataset.**

**
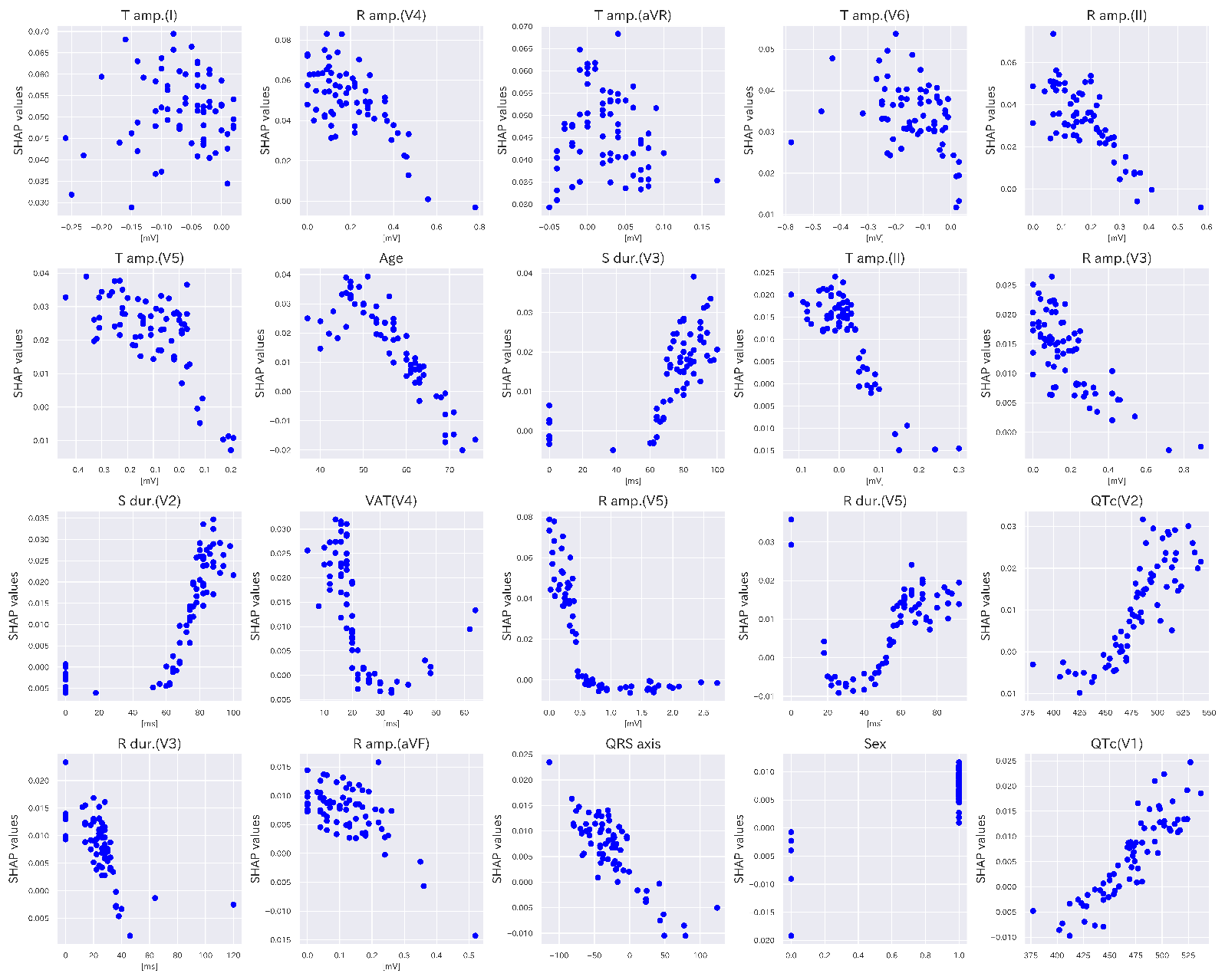
**

For the data included in cluster 4 on the external test dataset, scatter plots of 20 ECG parameters and SHAP values are shown in order of median SHAP value from top left to bottom right.

Abbreviations: SHAP, Shapley additive explanations; amp., amplitude; dur., duration; VAT, ventricular activation time; QTc, corrected QT interval.

**Supplementary Fig. 30. Scatter plots of ECG parameters and SHAP values in cluster 5 on the external test dataset.**

**
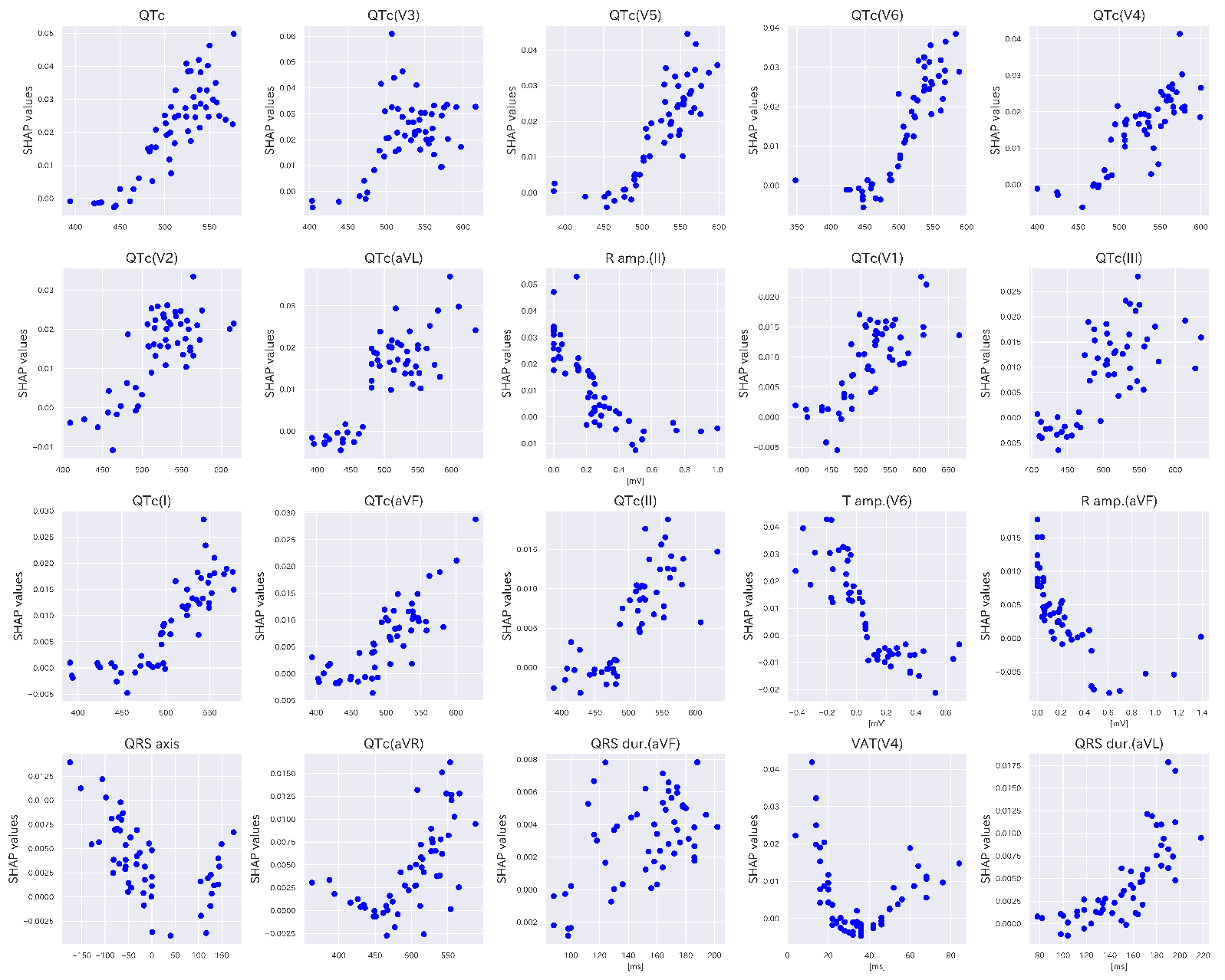
**

For the data included in cluster 5 on the external test dataset, scatter plots of 20 ECG parameters and SHAP values are shown in order of median SHAP value from top left to bottom right.

Abbreviations: SHAP, Shapley additive explanations; amp., amplitude; dur., duration; VAT, ventricular activation time; QTc, corrected QT interval.

**Supplementary Fig. 31. Scatter plots of ECG parameters and SHAP values in cluster 6 on the external test dataset.**

**
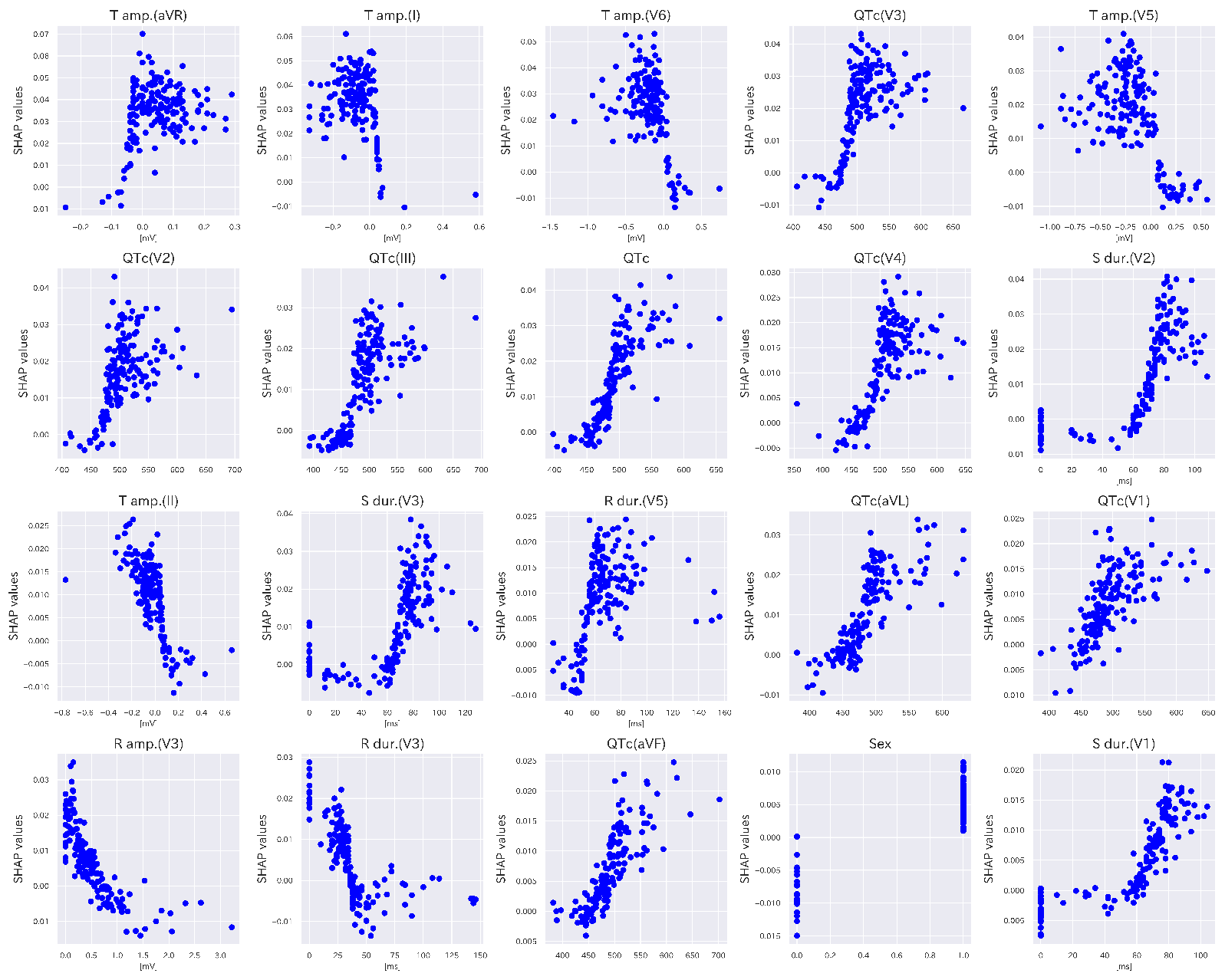
**

For the data included in cluster 6 on the external test dataset, scatter plots of 20 ECG parameters and SHAP values are shown in order of median SHAP value from top left to bottom right.

Abbreviations: SHAP, Shapley additive explanations; amp., amplitude; dur., duration; QTc, corrected QT interval.

**Supplementary Fig. 32. Scatter plots of ECG parameters and SHAP values in cluster 7 on the external test dataset.**

**
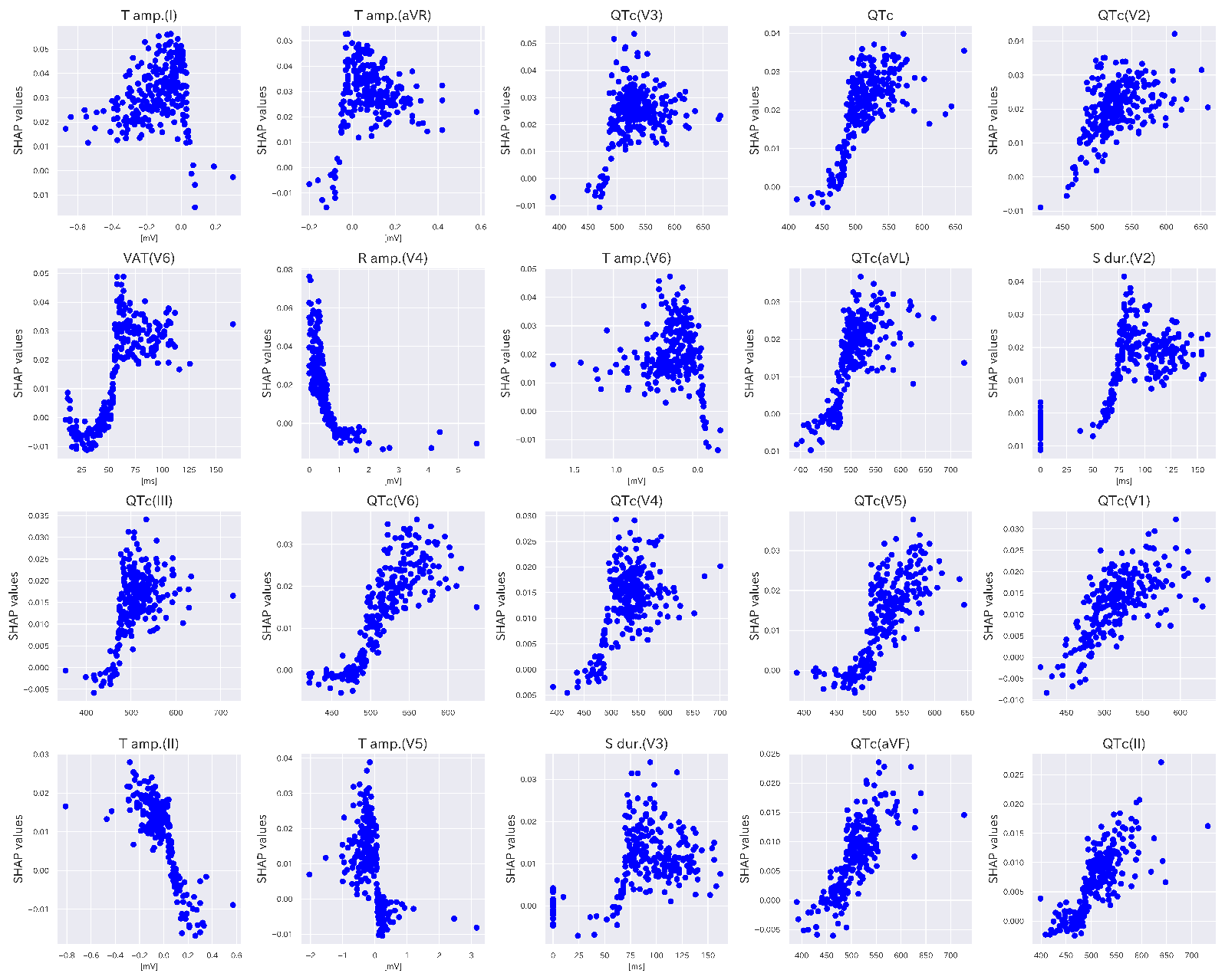
**

For the data included in cluster 7 on the external test dataset, scatter plots of 20 ECG parameters and SHAP values are shown in order of median SHAP value from top left to bottom right.

Abbreviations: SHAP, Shapley additive explanations; amp., amplitude; dur., duration; VAT, ventricular activation time; QTc, corrected QT interval.

**Supplementary Fig. 33. The categories of six ECG findings on a visualization of the clustered two-dimensional SHAP values for ECGs to predict reduced LVEF in the external test dataset.**

**
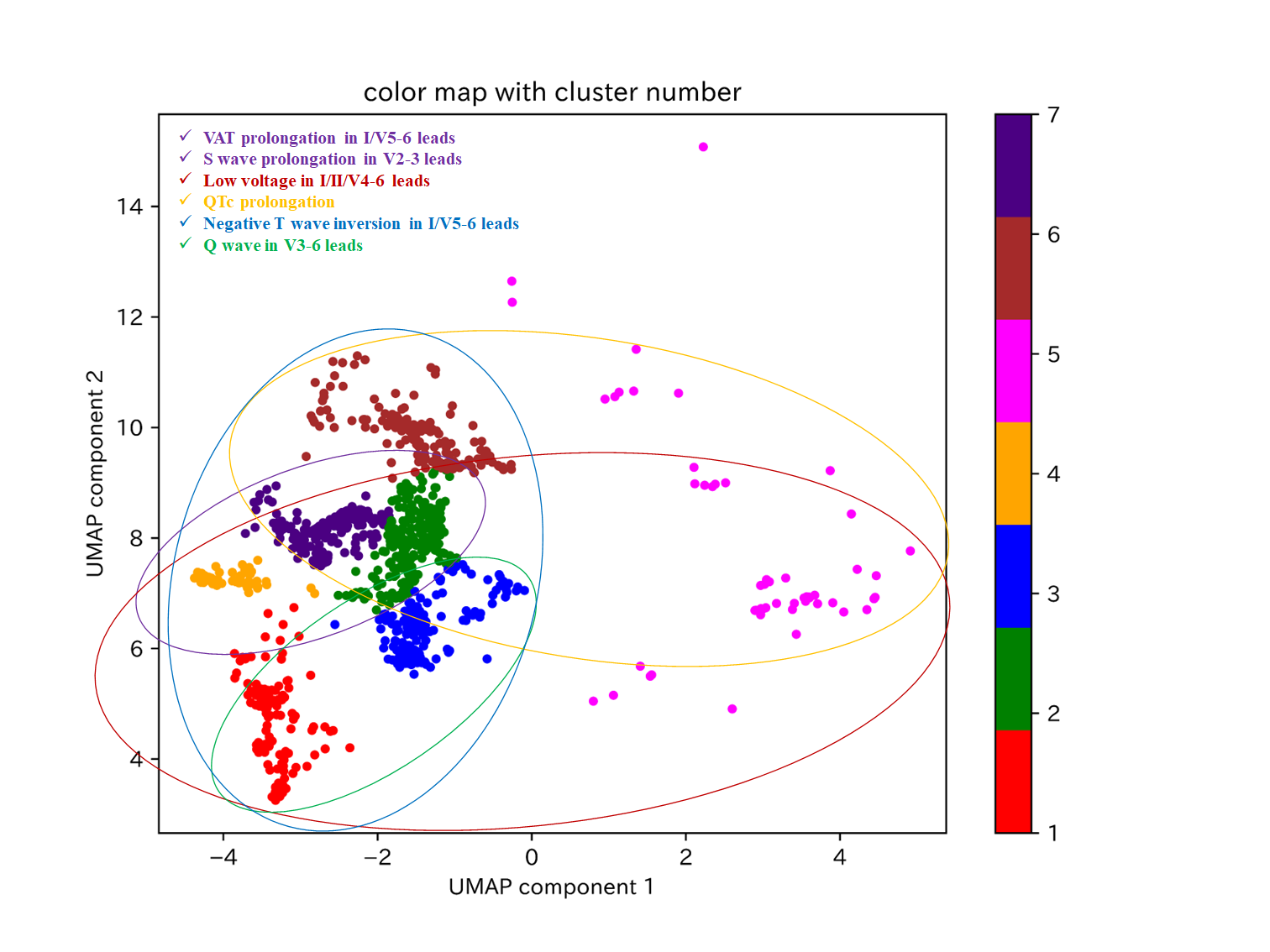
**

The purple circle represents the category of VAT prolongation in I/V5-6 leads and S wave prolongation in V2-3 leads. The brown circle represents the category of low voltage in I/II/V4-6 leads. The orange circle represents the category of QTc prolongation. The blue circle represents the category of negative T wave inversion in I/V5-6 leads. The green circle represents the category of Q wave in V3-6 leads.

Abbreviations: UMAP, uniform manifold approximation and projection; VAT, ventricular activation time; QTc, corrected QT interval.
